# Improved Method for Preparation of 3-(1*H*-Indol-3-yl)benzofuran-2(3*H*)-ones

**DOI:** 10.3390/molecules27061902

**Published:** 2022-03-15

**Authors:** Igor Yu. Grishin, Nikolai A. Arutiunov, Dmitrii A. Aksenov, Nicolai A. Aksenov, Alexander V. Aksenov, Amina Z. Gasanova, Elena A. Sorokina, Carolyn Lower, Michael Rubin

**Affiliations:** 1 Department of Chemistry and Pharmacy, North Caucasus Federal University, 1a Pushkin St., 355017 Stavropol, Russia; naarutiunov@ncfu.ru (N.A.A.); mr.twos@mail.ru (D.A.A.); naksenov@ncfu.ru (N.A.A.); aaksenov@ncfu.ru (A.V.A.); am.gasanova2017@gmail.com (A.Z.G.); 2 Organic Chemistry Department, Peoples’ Friendship University of Russia (RUDN University), 6 Miklukho-Maklaya St., 117198 Moscow, Russia; sorokina-ea@rudn.ru; 3 Department of Chemistry, University of Kansas, 1567 Irving Hill Road, Lawrence, KS 66045, USA; allisonwenger@ku.edu

**Keywords:** nitroalkanes, Brønsted acid catalysis, indoles, rearrangements, cascade transformations

## Abstract

3-(1*H*-Indol-3-yl)benzofuran-2(3*H*)-ones were efficiently accessed via polyphosphoric acid-mediated condensation of 3-(2-nitrovinyl)-1*H*-indoles with phenols.

## 1. Introduction

**Scheme 1:**
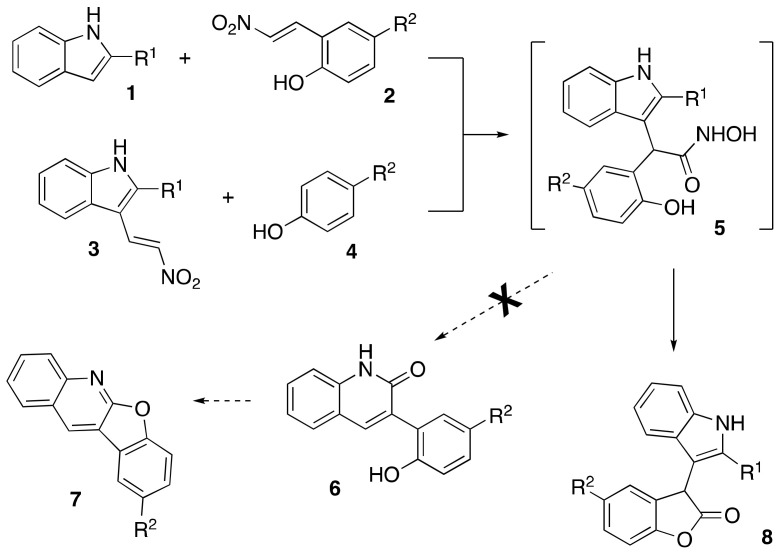
[4 + 1]-Spirocyclization of nitroalkenes and nitriles and subsequent rearrangements.

Since both indole and benzo–furanone structural fragments are omnipresent in nature and listed as privileged scaffolds for drug discovery, chimeric indigo-, or isoindigo-like molecules possessing both these moieties also attracted certain attention. There are several reports describing the preparation of these unique compounds and studies of their applications in medicinal chemistry and material sciences [1,2,3,4,5,6,7,8,9,10,11]. A common theme described in these reports is the metal-catalyzed C-C bond-forming reactions to accomplish the coupling between the indole and benzofuranone building blocks [1,5,8,10]. Other approaches used a Lewis acid-mediated reaction of indole derivatives with properly functionalized aromatic precursors (typically phenols or quinones) to furnish a five-membered lactone cycle in benzofurane [2,6,7]. We have recently established a synthetic approach towards benzofuro[2,3-b]quinoline cores employing a cascade transformation beginning with the condensation of indole and 1-methoxy-2-(2-nitrovinyl)benzenes in polyphosphoric acid (PPA). These studies ultimately resulted in a concise total synthesis of natural alkaloids norneocryptolepine and neocryptolepine [12,13]. During this investigation, the synthesis of benzofuro[2,3-b]quinoline **7** from 2-methylindole (**1**, R^1^ *=* Me) and 4-methyl-2-(2-nitrovinyl)phenol (**2**, R^2^ *=* Me) was attempted taking advantage of well-established protocol for PPA-mediated rearrangement of hydroxamic acid (**5**) into 2-quinolones (Scheme 1) [14,15,16]. However, the formation of compound **6** did not occur; instead, 3-(1*H*-Indol-3-yl)benzofuran-2(3*H*)-one **8** was formed in low yield, which was demonstrated on a single example [12]. We speculated that an alternative route towards the same compound could include the initial interaction of 3-(2-nitrovinyl)-1*H*-indoles (**3**) with phenols (**4**). Herein, we disclose an account of synthetic studies evaluating this idea and providing a reasonably efficient method for the preparation of 3-(1*H*-Indol-3-yl)benzofuran-2(3*H*)-ones **8** (Scheme 1).

## 2. Results and Discussion

It was anticipated that a Michael-like nucleophilic addition of phenol **4a** across an electron-deficient alkene moiety in nitrovinylindole **3a** would afford nitroalkane species **9aa**. A substituent placed in the para-position would ensure the installation of a new C-C bond in an ortho-position of the phenol. Under acidic conditions, compound **9aa** should exist in equilibrium with nitronate form, which is able to hydrolyze to afford hydroxamic acid of type **5** (Scheme 1). Subsequent intramolecular 5-*exo-trig* nucleophilic attack by phenol moiety with the release of hydroxylamine molecule would ensure the formation of the target structure **8aa**. To test this idea, equimolar amounts of indole **3a** and 4-chloro-3-methylphenol (**4a**) were subjected to reaction in 80% polyphosphoric acid at 80 °C, i.e., under conditions listed in our previous report [12]. The results were accurately reproduced, as compound **8aa** was obtained as the sole isolable product, albeit in marginal yield (Table 1, entry 1). The material balance of this reaction seems low, as most of the starting material decomposed to provide polymeric side products. Speculating that the reaction conditions were too harsh, an attempt was made to carry out the same transformation in the presence of a weaker Brønsted acid. These tests revealed that the acidity of formic acid was not sufficient to trigger the requisite tautomerization into nitronate. While the formation of nitroalkane **9aa** proceeded smoothly under these conditions, the subsequent transformation into **8aa** did not occur at any tested temperature (entries 2–4). An attempt to perform the reaction in a catalytic fashion in the presence of small quantities of Lewis (anhydrous zinc chloride, entry 5) or strong Brønsted acid (MsOH, entry 6) did not prove successful leading to the consumption of starting materials without any formation of the target product. An improvement in yield was observed when the reaction was carried out in MsOH at 80 and 60 °C, where product **8aa** was formed in 28% and 41% yield, respectively, while nitroalkane **9aa** was not observed (entries 7, 8). This demonstrated that lowering the temperature allowed for a positive effect on the reaction performance. Testing the reactions at 40 °C afforded an improved yield of **8aa**, though some unreacted nitroalkane **9aa** was also detected (entry 9). At room temperature, **9aa** was also formed, but its subsequent transformation into **8aa** did not occur (entry 10). Increasing mesic acid load (4 mL per 1 mmol of **3a**) also demonstrated a notable improvement in the reaction performance. Only nitroalkane **9aa** was formed at room temperature (entry 12), but at 40 °C it was not detected, while material **8aa** was formed as the sole product in 60% yield, as quantitated by ^1^H NMR. After isolation and purification on preparative scale, this translated into 55% yield (entry 11).

An attempt to carry out the process under microwave irradiation (isothermic mode) was also made, but the outcome of the reaction was not impressive (entry 13). Most likely, this result can be attributed to poor accuracy in maintaining low-temperature settings by our microwave equipment. Other strong acids were also tested as promoters of the featured transformation. In the presence of tosic acid, the formation of nitroalkane **9aa** in low yield was observed, while most starting material decomposed (entry 14). Triflic acid caused decomposition of the reaction mixture when used neat or in combination with CH_2_Cl_2_ as a co-solvent (entries 15, 16). Trifluoroacetic acid also caused decomposition, and no formation of the target product **8aa** was detected (entry 17).

**Scheme 2:**
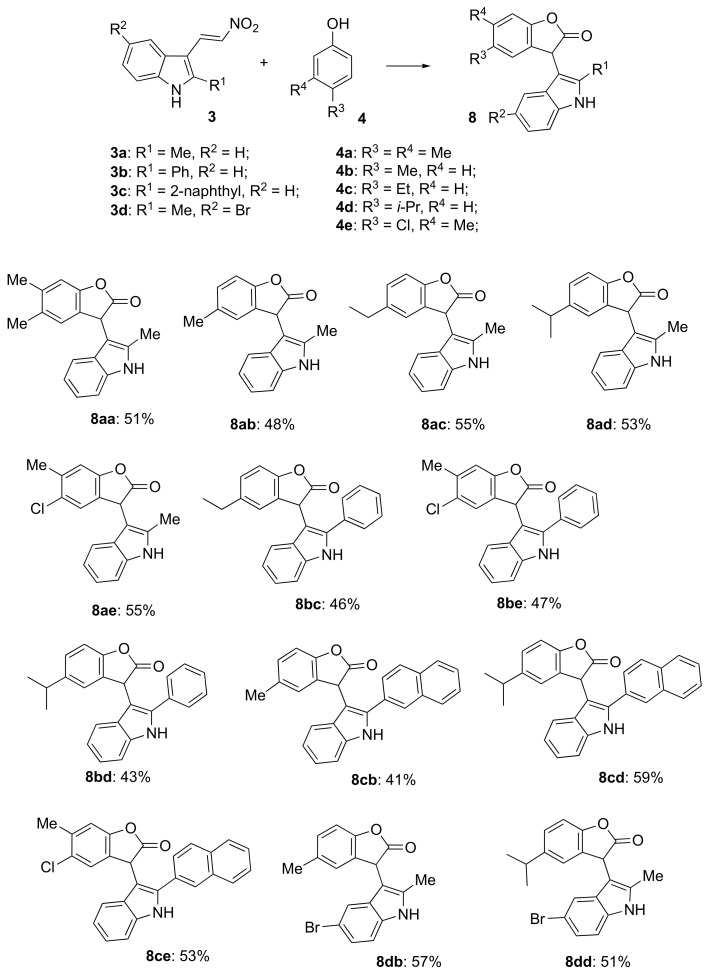
Preparation of for preparation of 3-(1*H*-Indol-3-yl)benzofuran-2(3*H*)-ones **8**.

**Figure 1:**
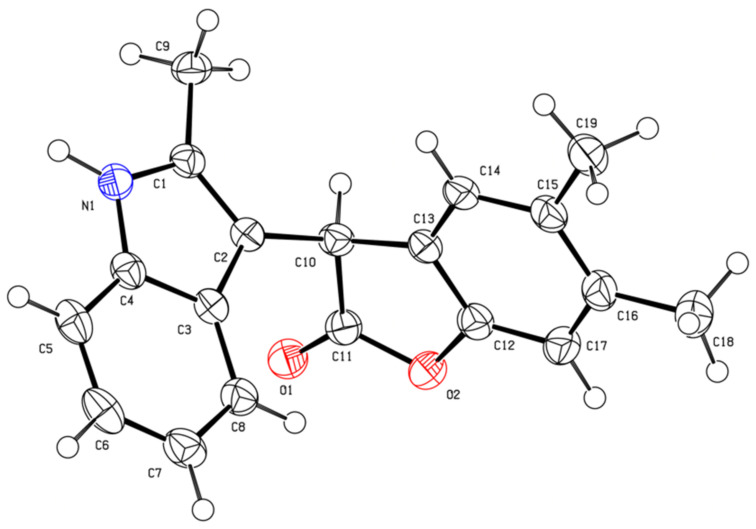
ORTEP drawing of X-ray structures of 5,6-dimethyl-3-(2-methyl-1*H*-indol-3-yl)benzofuran-2(3*H*)-one (**6aa**, CCDC #2154293). The thermal ellipsoids are shown at 50% probability.

With optimized conditions in hand, the synthesis of a small, focused library of analogs with a scope and limitation studies progressed, results are summarized in Scheme 2. The standard reaction conditions afforded a meaningful preparative yield across a large spectrum of substrates. Most substituents and functional groups tested (Alk, Ar, Hal) were well tolerated. Formation of 3-(1*H*-indol-3-yl)benzofuran-2(3*H*)-one core was unambiguously confirmed by X-ray crystallography of compound 8aa (Figure 1).

## 3. Conclusions

An improved protocol for preparation of 3-(1*H*-Indol-3-yl)benzofuran-2(3*H*)-ones **8** via Brønsted acid-mediated cascade reaction of 3-(2-nitrovinyl)-1*H*-indoles (**3**) with phenols (**4**) was developed. This transformation involves initial nucleophilic addition of phenol across electron-deficient alkene moiety followed by hydrolysis of nitroalkane **9** into hydroxamic acid **5** and subsequent intramolecular 5-*exo-trig* nucleophilic attack to furnish the lactone cycle. This methodology was employed to synthesize a small, focused library of target molecules. Investigation of the biological activity of these new 3-(1*H*-indol-3-yl)benzofuran-2(3*H*)-ones is currently underway in our laboratories.

## 4. Experimental Part

### General

NMR spectra (^1^H and ^13^C) were measured in solutions of CDCl_3_ or DMSO-*d_6_* on Bruker AVANCE-III HD instrument (Bruker Biospin AG, Faellanden, Switzerland) (at 400.40 or 100.61 MHz, respectively). HRMS spectra were measured in MeCN solutions on a Bruker maXis impact (Bruker Daltonik GmbH, Bremen, Germany) (electrospray ionization, employing HCO_2_Na–HCO_2_H for calibration) mass spectrometer. See Supplementary Materials for NMR (Figures S1–S30) and HRMS (Figures S31–S45) spectral charts and X-ray crystallography data (Figure S46). IR spectra were measured on an FT-IR spectrometer Shimadzu IRAffinity-1S (Shimadzu Europa GmbH, Duisburg, Germany) equipped with an ATR sampling module. Reaction progress, purity of isolated compounds, and R*_f_* values were assessed by TLC on Silufol UV-254 plates (Kavalier, Czech Republic). Column chromatography was performed on silica gel (32–63 μm, 60 Å pore size). Melting points were measured on a Stuart SMP30 apparatus. All reagents and solvents were purchased from commercial vendors and used as received.

(*E*)-*5-Bromo-2-methyl-3-(2-nitrovinyl)-1H-indole* (**3d**). This compound was prepared by a method described previously in the literature [17]. Red solid, mp (EtOH) 201–203 °C, R*_f_* 0.48 (EtOAc/Hex, 2:1). Yield: 2.24 g (8 mmol, 80%). ^1^H NMR (400 MHz, DMSO) δ 12.34 (s, 1H), 8.25 (d, *J =* 13.3 Hz, 1H), 8.05 (d, *J =* 1.8 Hz, 1H), 7.96 (d, *J =* 13.3 Hz, 1H), 7.40–7.27 (m, 2H), 2.58 (s, 3H); 13C NMR (101 MHz, DMSO) δ 148.3, 135.2, 132.6, 130.8, 127.1, 125.4, 122.3, 114.7, 113.7, 104.7, 12.1. IR, v_max_/cm^−1^: 3264, 1620, 1602, 1572, 1488, 1468, 1303, 1297, 1278, 1244, 1214, 1110. HRMS (ES TOF) calculated for (M + H)^+^ C_11_H_10_BrN_2_O_2_ 280.9920, found 280.9911 (3.4 ppm).

*Preparation of 3-(1H-indol-3-yl)benzofuran-2(3H)-ones* (**8aa**–**8dd**) *via Reaction of 3-(2-nitrovinyl)indoles with Phenols (General Procedure)*. A 5 mL round-bottomed flask equipped magnetic stirring bar was charged with 3-(2-nitrovinyl)indole (**3a**–**d**, 1 mmol), phenol (**4a**–**e**, 1.0 mmol), MeSO_3_H (4 mL). The mixture was vigorously stirred and heated to 40 °C for 1 h monitoring the reaction progress with TLC. After consumption of the starting 3-(2-nitrovinyl)indole, the reaction mass was neutralized with aqueous ammonia (25%) to pH 8 and extracted with ethyl acetate (3 × 25 mL). The combined organic extracts were washed with brine, dried with sodium sulphate, and concentrated in vacuum to obtain the crude material, which can be further purified by preparative column chromatography on silica gel.

*5,6-Dimethyl-3-(2-methyl-1H-indol-3-yl)benzofuran-2(3H)-one* (**8aa**). Colorless solid, mp (EtOH) 224.7–225.9 °C, R*_f_* 0.49 (EtOAc/Hex, 1:2). Yield: 148 mg (0.51 mmol, 51%). ^1^H NMR (400 MHz, Chloroform-*d*) δ 8.01 (s, 1H), 7.28–7.24 (m, 1H), 7.14–7.04 (m, 1H), 7.02 (s, 1H), 6.98–6.89 (m, 2H), 6.85 (s, 1H), 5.05 (s, 1H), 2.34 (s, 3H), 2.31 (s, 3H), 2.14 (s, 3H). ^13^C NMR (101 MHz, Chloroform-*d*) δ 177.0, 152.1, 137.7, 135.3, 133.6, 132.7, 127.1, 125.8, 124.8, 121.7, 120.0, 117.8, 111.8, 110.7, 105.6, 41.5, 20.5, 19.6, 12.0. IR, v_max_/cm^−1^: 3372, 3101, 3053, 1779, 1491, 1455, 1302, 1238, 1115. HRMS (ES TOF) calculated for (M + Na)^+^ C_19_H_17_NNaO_2_ 314.1151, found 314.1149 (0.8 ppm).

*5-Methyl-3-(2-methyl-1H-indol-3-yl)benzofuran-2(3H)-one* (**8ab**). Colorless solid, mp (EtOH) 175–175 °C, R*_f_* 0.3 (EtOAc/Hex, 1:4). Yield: 134 mg (0.48 mmol, 48%). ^1^H NMR (400 MHz, CDCl_3_) δ 8.10 (s, 1H), 7.25 (d, *J =* 7.6 Hz, 1H), 7.16–7.07 (m, 3H), 6.93 (m, 3H), 5.08 (s, 1H), 2.27 (s, 3H), 2.25 (s, 3H). ^13^C NMR (101 MHz, CDCl_3_) δ 176.9, 151.8, 135.3, 134.2, 133.8, 129.5, 127.7, 126.9, 125.6, 121.7, 119.9, 117.7, 110.8, 110.4, 105.2, 41.7, 21.2, 11.8.IR, v_max_/cm^−1^:3364, 2974, 2934, 1817, 1789, 1620, 1480, 1465, 1307, 1230, 1132. HRMS (ES TOF) calculated for (M + Na)^+^ C_18_H_15_NNaO_2_ 300.0995, found 300.0984 (3.8 ppm).

*5-Ethyl-3-(2-methyl-1H-indol-3-yl)benzofuran-2(3H)-one* (**8ac**). Colorless viscous liquid, R*_f_* 0.51 (EtOAc/Hex, 1:2). Yield: 160 mg (0.55 mmol, 55%). ^1^H NMR (400 MHz, Chloroform-*d*) δ 8.10 (s, 1H), 7.25 (d, *J =* 5.6 Hz, 1H), 7.21–7.11 (m, 2H), 7.09 (t, *J =* 7.9 Hz, 1H), 6.94 (d, *J =* 7.2 Hz, 3H), 5.09 (s, 1H), 2.55 (q, *J =* 7.6 Hz, 2H), 2.27 (s, 3H), 1.15 (t, *J =* 7.6 Hz, 3H). ^13^C NMR (101 MHz, Chloroform-*d*) δ 176.9, 151.9, 140.8, 135.3, 133.8, 128.3, 127.7, 127.0, 124.5, 121.6, 119.9, 117.7, 110.7, 110.5, 105.2, 41.7, 28.6, 16.0, 11.8. IR, v_max_/cm^−1^: 3396, 3061, 2966, 1813, 1783, 1733, 1481, 1461, 1242, 1135. HRMS (ES TOF) calculated for (M + Na)^+^ C_19_H_17_NNaO_2_ 314.1151, found 314.1150 (0.5 ppm).

*5-Isopropyl-3-(2-methyl-1H-indol-3-yl)benzofuran-2(3H)-one* (**8ad**). Yellow oil, R*_f_* 0.3 (EtOAc/Hex, 1:4). Yield: 162 mg (0.53 mmol, 53%). ^1^H NMR (400 MHz, Chloroform-*d*) δ 8.04 (s, 1H), 7.28 (s, 1H), 7.23–7.17 (m, 1H), 7.14 (d, *J =* 8.3 Hz, 1H), 7.09 (t, *J =* 8.0 Hz, 1H), 6.99–6.74 (m, 3H), 5.11 (s, 1H), 2.95–2.66 (m, 1H), 2.33 (s, 3H), 1.16 (t, *J =* 7.2 Hz, 6H).^13^C NMR (101 MHz, CDCl_3_) δ 176.7, 152.0, 145.5, 135.3, 133.7, 127.5, 127.2, 126.9, 123.2, 121.7, 120.0, 117.8, 110.7, 110.5, 105.3, 41.7, 34.0, 24.5, 24.1, 12.0. IR, v_max_/cm^−1^: 3369, 2976, 2937, 1812, 1776, 1629, 1455, 1323, 1237, 1139. HRMS (ES TOF) calculated for (M + Na)^+^ C_20_H_19_NNaO_2_ 328.1308, found 328.1295 (3.8 ppm).

*5-Chloro-6-methyl-3-(2-methyl-1H-indol-3-yl)benzofuran-2(3H)-one* (**8ae**). Yellow oil, R*_f_* 0.28 (EtOAc/Hex, 1:4). Yield: 172 mg (0.55 mmol, 55%). ^1^H NMR (400 MHz, Chloroform-*d*) δ 8.06 (s, 1H), 7.28 (s, 1H), 7.17–7.06 (m, 3H), 6.96 (d, *J =* 7.3 Hz, 1H), 6.90 (d, *J =* 11.0 Hz, 1H), 5.08 (s, 1H), 2.43 (s, 3H), 2.32 (s, 3H). ^13^C NMR (101 MHz, Chloroform-*d*) δ 175.9, 152.3, 137.4, 135.3, 133.8, 129.9, 126.8, 126.7, 125.5, 121.9, 120.2, 117.6, 113.1, 110.8, 104.8, 41.5, 20.8, 12.0. IR, v_max_/cm^−1^: 3403, 2962, 1811, 1787, 1622, 1482, 1459, 1299, 1230, 1138, 1069, 1051, 906, 733. HRMS (ES TOF) calculated for (M + H)^+^ C_18_H_15_ClNO_2_ 312.0786, found 312.0792 (−2.1 ppm).

*5-Ethyl-3-(2-phenyl-1H-indol-3-yl)benzofuran-2(3H)-one* (**8bc**). Colorless solid, mp (EtOH) 201.1–203.3 °C, R*_f_* 0.68 (EtOAc/Hex, 1:2). Yield: 162 mg (0.46 mmol, 46%). ^1^H NMR (400 MHz, Chloroform-*d*) δ 8.29 (s, 1H), 7.82–7.66 (m, 2H), 7.56–7.42 (m, 3H), 7.38 (d, *J =* 8.1 Hz, 1H), 7.17 (d, *J =* 8.2 Hz, 3H), 7.00–6.86 (m, 2H), 6.76 (s, 1H), 5.28 (s, 1H), 2.52 (q, *J =* 7.6 Hz, 2H), 1.11 (t, *J =* 7.6 Hz, 3H). ^13^C NMR (101 MHz, Chloroform-*d*) δ 176.8, 151.9, 140.8, 138.3, 136.0, 132.0, 129.3 (2C), 128.9, 128.7 (2C), 128.4, 127.6, 127.0, 124.6, 122.9, 120.5, 119.1, 111.3, 110.5, 106.0, 42.1, 28.6, 16.0. IR, v_max_/cm^−1^: 3432, 3050, 2927, 1815, 1618, 1487, 1457, 1310, 1270, 1236, 1137, 1049. HRMS (ES TOF) calculated for (M + Na)^+^ C_24_H_19_NNaO_2_ 376.1308, found 376.1319 (−3.0 ppm).

*5-Isopropyl-3-(2-phenyl-1H-indol-3-yl)benzofuran-2(3H)-one* (**8bd**). Colorless solid, mp (EtOH) 212.5–214.2 °C, R*_f_* 0.63 (EtOAc/Hex, 1:2). Yield: 158 mg (0.43 mmol, 43%). ^1^H NMR (400 MHz, Chloroform-*d*) δ 8.30 (s, 1H), 7.73 (s, 2H), 7.47 (dt, *J =* 24.5, 7.4 Hz, 3H), 7.38 (d, *J =* 8.2 Hz, 1H), 7.24–7.11 (m, 3H), 7.00–6.86 (m, 2H), 6.73 (s, 1H), 5.29 (s, 1H), 2.88–2.72 (m, 1H), 1.13 (d, *J =* 6.9 Hz, 3H), 1.11 (d, *J =* 6.9 Hz, 3H). ^13^C NMR (101 MHz, Chloroform-*d*) δ 176.9, 152.0, 145.5, 138.3, 136.0, 132.0, 129.3 (2C), 129.0, 128.7 (2C), 127.5, 127.0, 126.9, 123.3, 122.8, 120.4, 119.1, 111.2, 110.5, 105.9, 42.1, 33.9, 24.4, 24.1. IR, v_max_/cm^−1^ 3420, 3061, 2974, 1809, 1483, 1449, 1240, 1138, 1047. HRMS (ES TOF) calculated for (M + Na)^+^ C_25_H_21_NNaO_2_ 390.1464, found 390.1468 (−0.9 ppm).

*5-Chloro-6-methyl-3-(2-phenyl-1H-indol-3-yl)benzofuran-2(3H)-one* (**8be**). Colorless solid, mp (EtOH) 186.2–188.2 °C, R*_f_* 0.69 (EtOAc/Hex, 1:2). Yield: 175 mg (0.47 mmol, 47%). ^1^H NMR (400 MHz, Chloroform-*d*) δ 8.33 (s, 1H), 7.70 (d, *J =* 7.4 Hz, 2H), 7.56–7.43 (m, 3H), 7.39 (d, *J =* 8.1 Hz, 1H), 7.21–7.15 (m, 1H), 7.13 (s, 1H), 7.04 (s, 1H), 6.96 (t, *J =* 7.6 Hz, 1H), 6.76 (d, *J =* 7.9 Hz, 1H), 5.26 (s, 1H), 2.43 (s, 3H). ^13^C NMR (101 MHz, Chloroform-*d*) δ 176.1, 152.3, 138.5, 137.5, 136.0, 131.7, 129.9, 129.4 (2C), 129.1, 128.7 (2C), 126.7, 126.7, 125.6, 123.0, 120.6, 118.8, 113.1, 111.4, 105.3, 41.9, 20.9. IR, v_max_/cm^−1^: 3431, 3053, 2926, 1807, 1473, 1453, 1399, 1379, 1264, 1183. HRMS (ES TOF) calculated for (M + Na)^+^ C_23_H_16_ClNNaO_2_ 396.0762, found 396.0759 (0.6 ppm).

*5-Methyl-3-(2-(naphthalen-2-yl)-1H-indol-3-yl)benzofuran-2(3H)-one* (**8cb**). Colorless viscous liquid, R*_f_* 0.66 (EtOAc/Hex, 1:2). Yield: 159 mg (0.41 mmol, 41%). ^1^H NMR (400 MHz, Chloroform-*d*) δ 8.40 (s, 1H), 8.18 (s, 1H), 7.96 (d, *J =* 8.4 Hz, 1H), 7.90 (dd, *J =* 6.3, 3.3 Hz, 2H), 7.80 (d, *J =* 7.7 Hz, 1H), 7.61–7.53 (m, 2H), 7.39 (d, *J =* 8.2 Hz, 1H), 7.21–7.15 (m, 1H), 7.13 (s, 2H), 6.96 (t, *J =* 7.5 Hz, 1H), 6.90 (s, 1H), 6.81 (s, 1H), 5.37 (s, 1H), 2.22 (s, 3H). ^13^C NMR (101 MHz, Chloroform-*d*) δ 176.9, 151.8, 138.3, 136.2, 134.2, 133.5, 133.2, 129.6, 129.3, 129.1, 128.4, 128.0, 127.9, 127.7, 127.1, 127.0, 126.9, 126.1, 125.7, 123.0, 120.5, 119.0, 111.3, 110.5, 106.4, 42.1, 21.2. IR, v_max_/cm^−1^: 3372, 3065, 2986, 1809, 1783, 1735, 1489, 1242, 1140. HRMS (ES TOF) calculated for (M + Na)^+^ C_27_H_19_NNaO_2_ 412.1308, found 412.1309 (−0.3 ppm).

*5-Isopropyl-3-(2-(naphthalen-2-yl)-1H-indol-3-yl)benzofuran-2(3H)-one* (**8cd**). Colorless solid, mp (EtOH) 21–216 °C, R*_f_* 0.48 (EtOAc/Hex, 1:4). Yield: 246 mg (0.3 mmol, 59%). ^1^H NMR (400 MHz, Chloroform-*d*) δ 8.41 (s, 1H), 8.20 (s, 1H), 7.92 (m, 4H), 7.55 (dd, *J =* 6.3, 3.2 Hz, 2H), 7.40 (d, *J =* 8.2 Hz, 1H), 7.17 (q, *J =* 7.5 Hz, 3H), 6.94 (m, 2H), 6.81 (s, 1H), 5.38 (s, 1H), 2.93–2.61 (m, 1H), 1.10 (dd, *J =* 9.7, 6.8 Hz, 6H). ^13^C NMR (101 MHz, Chloroform-*d*) δ 177.0, 152.0, 145.5, 138.3, 136.2, 133.5, 133.2, 129.3, 129.1, 128.4, 128.0, 127.9, 127.5, 127.1, 127.90, 126.9, 126.9, 126.1, 123.3, 122.9, 120.5, 119.1, 111.3, 110.5, 106.3, 42.2, 33.9, 24.4, 24.1. IR, v_max_/cm^−1^: 3418, 3078, 2936, 1845, 1612, 1456, 1399, 1365, 1323, 1214, 1178. HRMS (ES TOF) calculated for (M + H)^+^ C_29_H_24_NO_2_ 418.1802, found 418.1793 (2.0 ppm).

*5-Chloro-6-methyl-3-(2-(naphthalen-2-yl)-1H-indol-3-yl)benzofuran-2(3H)-one* (**8ce**). Colorless solid, mp (EtOH) 218–219 °C, R*_f_* 0.48 (EtOAc/Hex, 1:4). Yield: 224 mg (0.53 mmol, 53%). ^1^H NMR (400 MHz, Chloroform-*d*) δ 8.50 (s, 1H), 8.14 (s, 1H), 7.96–7.84 (m, 3H), 7.75 (d, *J =* 8.7 Hz, 1H), 7.55 (dt, *J =* 6.4, 3.5 Hz, 2H), 7.38–7.35 (m, 1H), 7.20–7.16 (m, 1H), 7.11 (s, 1H), 7.05 (d, *J =* 1.3 Hz, 1H), 6.98 (t, *J =* 7.6 Hz, 1H), 6.83 (s, 1H), 5.36 (s, 1H), 2.41 (s, 3H). ^13^C NMR (101 MHz, Chloroform-*d*) δ 176.3, 152.2, 147.4, 138.4, 137.4, 136.1, 133.4, 133.2, 130.4, 129.9, 129.1, 128.4, 127.9, 127.0, 126.6, 126.0, 125.5, 124.4, 123.0, 120.7, 120.6, 118.7, 113.0, 111.5, 105.6, 42.0, 20.8. IR, v_max_/cm^−1^: 3415, 3065, 2926, 1805, 1628, 1606, 1474, 1455, 1397, 1371, 1347, 1254, 1178, 1061, 906. HRMS (ES TOF) calculated for (M + Na)^+^ C_27_H_18_NNaO_2_ 446.0918, found 446.0909 (2.2 ppm).

*3-(5-Bromo-2-methyl-1H-indol-3-yl)-5-methylbenzofuran-2(3H)-one* (**8db**). Yellowish viscous liquid, R*_f_* 0.34 (EtOAc/Hex, 1:2). Yield: 202 mg (0.57 mmol, 57%). ^1^H NMR (400 MHz, Chloroform-*d*) δ 8.21 (s, 1H), 7.18–7.03 (m, 5H), 6.90–6.84 (m, 1H), 5.02 (s, 1H), 2.26 (s, 3H), 2.19 (s, 3H). ^13^C NMR (101 MHz, Chloroform-*d*) δ 176.8, 151.6, 135.2, 134.5, 133.9, 129.8, 128.7, 127.1, 125.4, 124.5, 120.1, 113.2, 112.2, 110.6, 104.9, 41.5, 21.2, 11.8. IR, v_max_/cm^−1^: 3376, 2998, 2898, 1813, 1779, 1737, 1485, 1463, 1220, 1131. HRMS (ES TOF) calculated for (M + H)^+^ C_18_H_15_BrNO_2_ 356.0281, found 356.0282 (−0.4 ppm).

*3-(5-Bromo-2-methyl-1H-indol-3-yl)-5-isopropylbenzofuran-2(3H)-one* (**8dd**). Colorless viscous liquid, R*_f_* 0.43 (EtOAc/Hex, 1:2). Yield: 195 mg (0.51 mmol, 51%). ^1^H NMR (400 MHz, Chloroform-*d*) δ 8.10 (s, 1H), 7.23 (dd, *J =* 8.3, 2.0 Hz, 1H), 7.20–7.09 (m, 3H), 6.95 (t, *J =* 1.6 Hz, 2H), 5.06 (s, 1H), 2.95–2.77 (m, 1H), 2.31 (s, 3H), 1.19 (d, *J =* 6.1 Hz, 3H), 1.17 (d, *J =* 6.2 Hz, 3H). ^13^C NMR (101 MHz, Chloroform-*d*) δ 176.4, 151.9, 145.6, 135.1, 133.8, 128.8, 127.2, 126.8, 124.4, 123.0, 120.3, 113.1, 112.0, 110.7, 104.8, 41.5, 33.9, 24.3, 24.1, 12.0. IR, v_max_/cm^−1^: 3392, 2970, 1813, 1789, 1732, 1484, 1242, 1045. HRMS (ES TOF) calculated for (M + Na)^+^ C_20_H_18_BrNNaO_2_ 406.0413, found 406.0412 (0.3 ppm).

*Preparation of 4,5-dimethyl-2-(1-(2-methyl-1H-indol-3-yl)-2-nitroethyl)phenol (**9aa**) via Reaction of 2-methyl-3-(2-nitrovinyl)indole with 3,4-dimethylphenol* (**4ae**). A 5 mL round-bottomed flask equipped magnetic stirring bar was charged with 2-methyl-3-(2-nitrovinyl)indole (**3a**, 1 mmol), 3,4-dimethylphenol (**4a**, 1.0 mmol), MeSO_3_H (4 mL). The mixture was vigorously stirred and heated to 20 °C for 1 h monitoring the reaction progress with TLC. After consumption of the starting 2-methyl-3-(2-nitrovinyl)indole, the reaction mass was neutralized with aqueous ammonia (25%) to pH 8 and extracted with ethyl acetate (3 × 25 mL). The combined organic extracts were washed with brine, dried with sodium sulphate, and concentrated in vacuum to obtain the crude material, which can be further purified by preparative column chromatography on silica gel.

Yellowish viscous liquid, R*_f_* 0.43 (EtOAc/Hex, 1:2). Yield: 172 mg (0.53 mmol, 53%). ^1^H NMR (400 MHz, Chloroform-*d*) δ 7.88 (s, 1H), 7.50 (d, *J =* 7.8 Hz, 1H), 7.24–7.20 (m, 1H), 7.12 (ddd, *J =* 8.0, 7.1, 1.3 Hz, 1H), 7.09–7.02 (m, 2H), 6.52 (s, 1H), 5.40–5.22 (m, 2H), 5.09 (dd, *J =* 12.0, 9.1 Hz, 1H), 2.35 (s, 3H), 2.14 (s, 3H), 2.14 (s, 3H). ^13^C NMR (101 MHz, Chloroform-*d*) δ 151.3, 136.9, 135.5, 133.5, 128.9, 128.8, 127.1, 122.6, 121.4, 119.8, 118.7, 117.7, 110.9, 107.5, 77.7, 35.4, 19.5, 19.2, 12.0. IR, v_max_/cm^−1^: 3399, 2982, 1735, 1706, 1548, 1461, 1372, 1242. HRMS (ES TOF) calculated for (M + Na)^+^ C_19_H_20_N_2_NaO_3_ 347.1366, found 347.1367 (−0.3 ppm).

## Figures and Tables

**Table 1:**
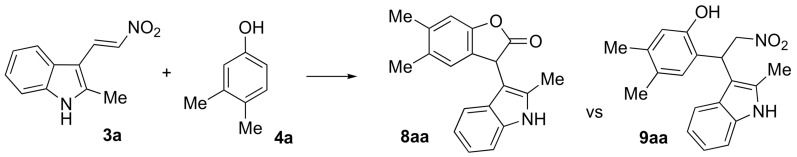
Optimization of the reaction conditions for a direct conversion of 3-(2-nitrovinyl)-1*H*-indole **3a** and phenol **4a** into 3-(1*H*-Indol-3-yl)benzofuran-2(3*H*)-ones **8aa**.

	Acid (Volume in mL per mmol) ^a^	Temperature (°C)	Yield of 8aa, % ^b^	Yield of 9aa, % ^b^
1	PPA, 80% (2)	80	15	-
2	HCOOH (2)	80	-	50
3	HCOOH (2)	40	-	40
4	HCOOH (2)	20	-	30
5	Cat. ZnCl_2_ in EtOH ^c^	80	-	-
6	Cat. MsOH in EtOH ^c^	80	-	-
7	MsOH (2)	80	28	0
8	MsOH (2)	60	41	0
9	MsOH (2)	40	45	10
10	MsOH (2)	20	0	50
**11**	**MsOH (4)**	**40**	**60 (55)** ^d^	**0**
12	MsOH (4)	20	0	53
13	MsOH (4) ^e^	40 ^e^	34	12
14	TsOH ^f^	40	0 ^h^	31
15	TfOH (4)	40	0 ^h^	0
16	TfOH (2) ^g^	40	0 ^h^	0
17	CF_3_COOH (4)	40	0 ^h^	0

^a^ Volume of acid in mL per 1 mmol of starting material **3a** is listed. ^b^ NMR yields are reported unless specified otherwise. The best result is shown in bold. ^c^ 15 mg of ZnCl_2_ or 40 µL of MsOH in 2 mL of EtOH. Decomposition of the reaction mixture was observed due to polymerization. No low molecular mass products were detected. ^d^ Isolated yield of purified product **8aa** is provided in parentheses. ^e^ The mixture was heated in microwave reactor maintaining constant temperature program. ^f^ 1 g of TsOH in 1 mL of CH_2_Cl_2_ was used. ^g^ In 2 mL of CH_2_Cl_2_. ^h^ Decomposition of the reaction mixture. No target product was detected.

## Data Availability

Supplementary Materials include NMR spectral charts.

## Supplementary Materials

The following are available online at https://www.mdpi.com/article/10.3390/molecules27061902/s1, ^1^H and ^13^C NMR spectral charts.

## Sample Availability

Not available.
