# Supplementary material for: Improved Method for Preparation of 3-(1H-Indol-3-yl)benzofuran-2(3H)-ones
Source: Molecules. 2022 Mar 15;27(6):1902. doi: 10.3390/molecules27061902 (PMC8950327; doi:10.3390/molecules27061902)
Supplement: Supplementary file 1 [file molecules-27-01902-s001.zip › suppl/!!!SI í¬ Letter v5.pdf]

Electronic Supplementary Material (ESI)

Improved method for preparation of 3-(1H-Indol-3-yl)benzofuran-2(3H)-ones

Igor Yu. Grishin,[1,*] Nikolai A. Arutiunov [1], Dmitrii A. Aksenov [1], Nicolai A. Aksenov [1], Alexander V. Aksenov [1], Amina Z. Gasanova [1], Elena A. Sorokina [2], Carolyn Lower [3] and Michael Rubin [1,3,*]

1 Department of Chemistry, North Caucasus Federal University, 1a Pushkin St., 355017 Stavropol, Russia; naksenov@ncfu.ru (N.A.A.); naarutiunov@ncfu.ru (N.A.A.); mr.twos@mail.ru (D.A.A.); aaksenov@ncfu.ru (A.V.A.); am.gasanova2017@gmail.com (A.Z.G.)
2 Organic Chemistry Department, Peoples' Friendship University of Russia (RUDN University),
6, Miklukho-Maklaya St., 117198 Moscow, Russia; sorokina-ea@rudn.ru
3 Department of Chemistry, University of Kansas, 1567 Irving Hill Road, Lawrence, KS 66045, USA; allisonwenger@ku.edu

Supporting Information

NMR Spectral Charts........ S2
^{1}H and ^{13}C NMR spectral charts for (*E*)-5-bromo-2-methyl-3-(2-nitrovinyl)-1*H*-indole (3d)……….. S2
^{1}H and ^{13}C NMR spectral charts for 3-(1*H*-indol-3-yl)benzofuran-2(3*H*)-ones (8aa-8dd)………. S4
^{1}H and ^{13}C NMR spectral charts for 4,5-Dimethyl-2-(1-(2-methyl-1*H*-indol-3-yl)-2-nitroethyl)phenol (9aa):……….. S30
HRMS spectral charts………. S32
HRMS spectral chart for starting (E)-5-bromo-2-methyl-3-(2-nitrovinyl)-1H-indole (3d)……….. S32
HRMS spectral charts for 3-(1*H*-indol-3-yl)benzofuran-2(3*H*)-ones (8aa-8dd)………. S33
HRMS spectral chart for 4,5-Dimethyl-2-(1-(2-methyl-1*H*-indol-3-yl)-2-nitroethyl)phenol (9aa):………. S33
X-Ray crystallography data………. S39
References........ S47

^{1}H and ^{13}C NMR spectral charts for (*E*)-5-bromo-2-methyl-3-(2-nitrovinyl)-1*H*-indole (3d)

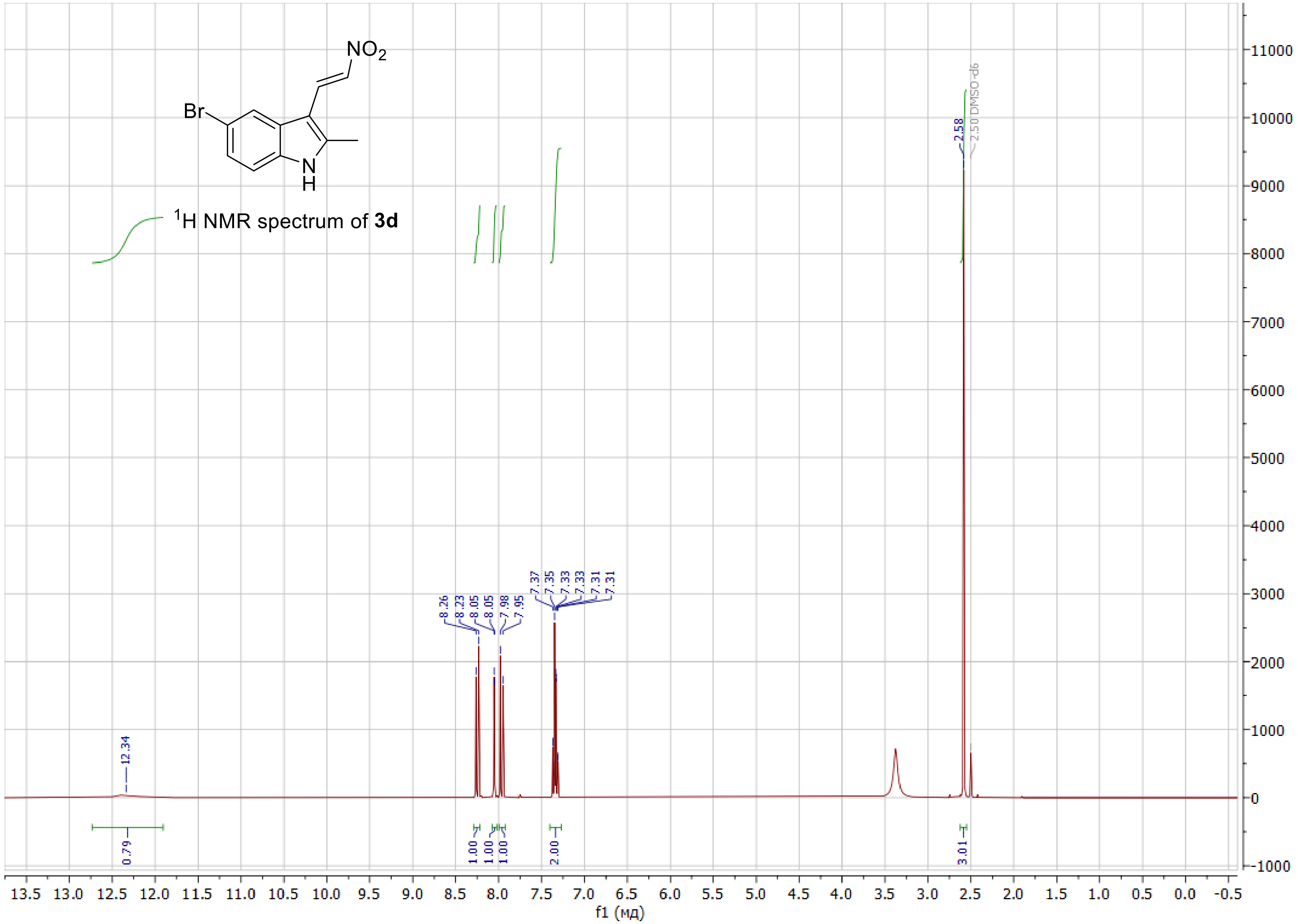

Figure S1. ^{1}H NMR spectrum of **3d**

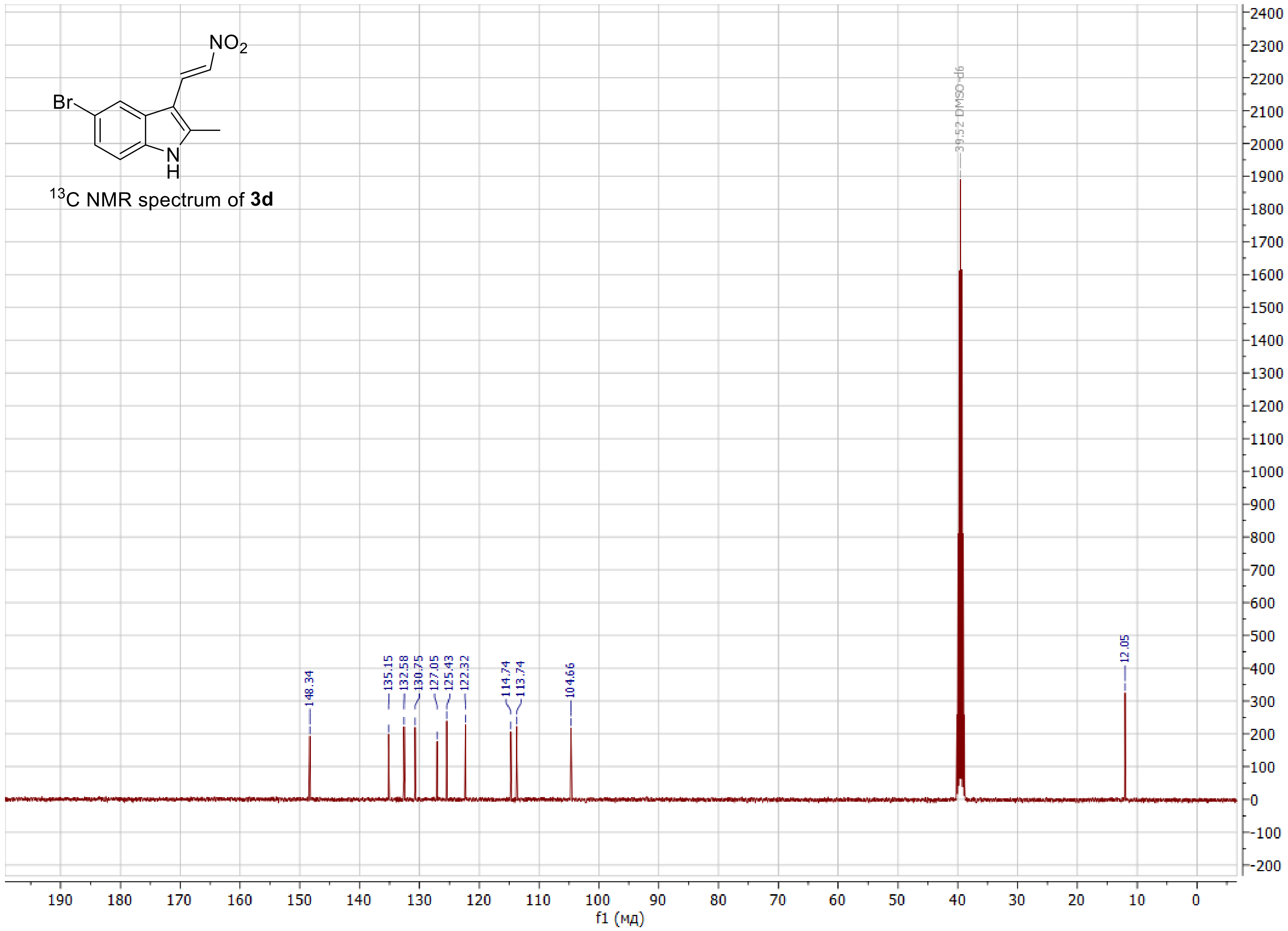

Figure S2. ^{13}C NMR spectrum of **3d**

^{1}H and ^{13}C NMR spectral charts for 3-(1*H*-indol-3-yl)benzofuran-2(3*H*)-ones (8aa-8dd)

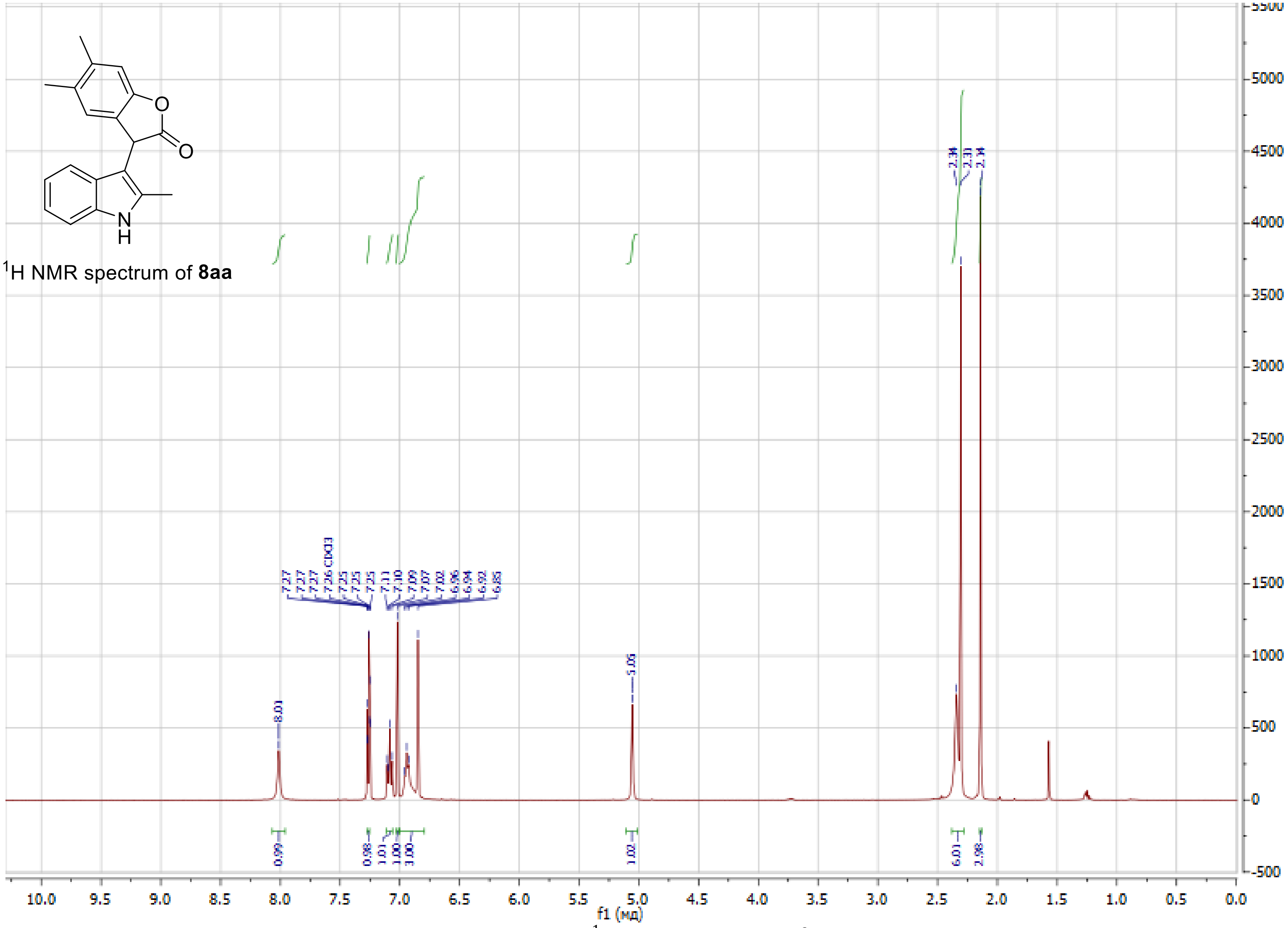

Figure S3. ^{1}H NMR spectrum of **8aa**

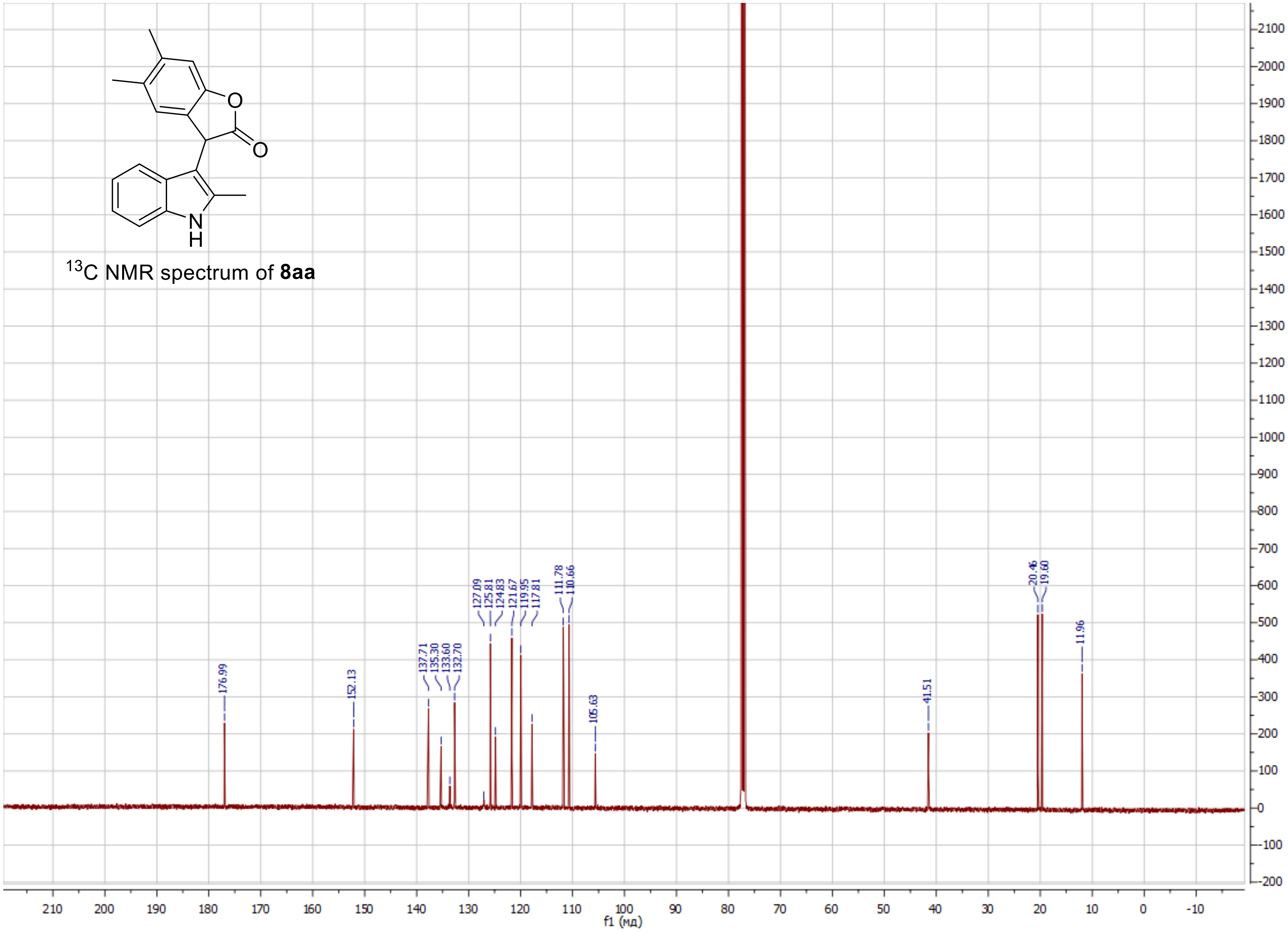

Figure S4. ^{13}C NMR spectrum of **8aa**

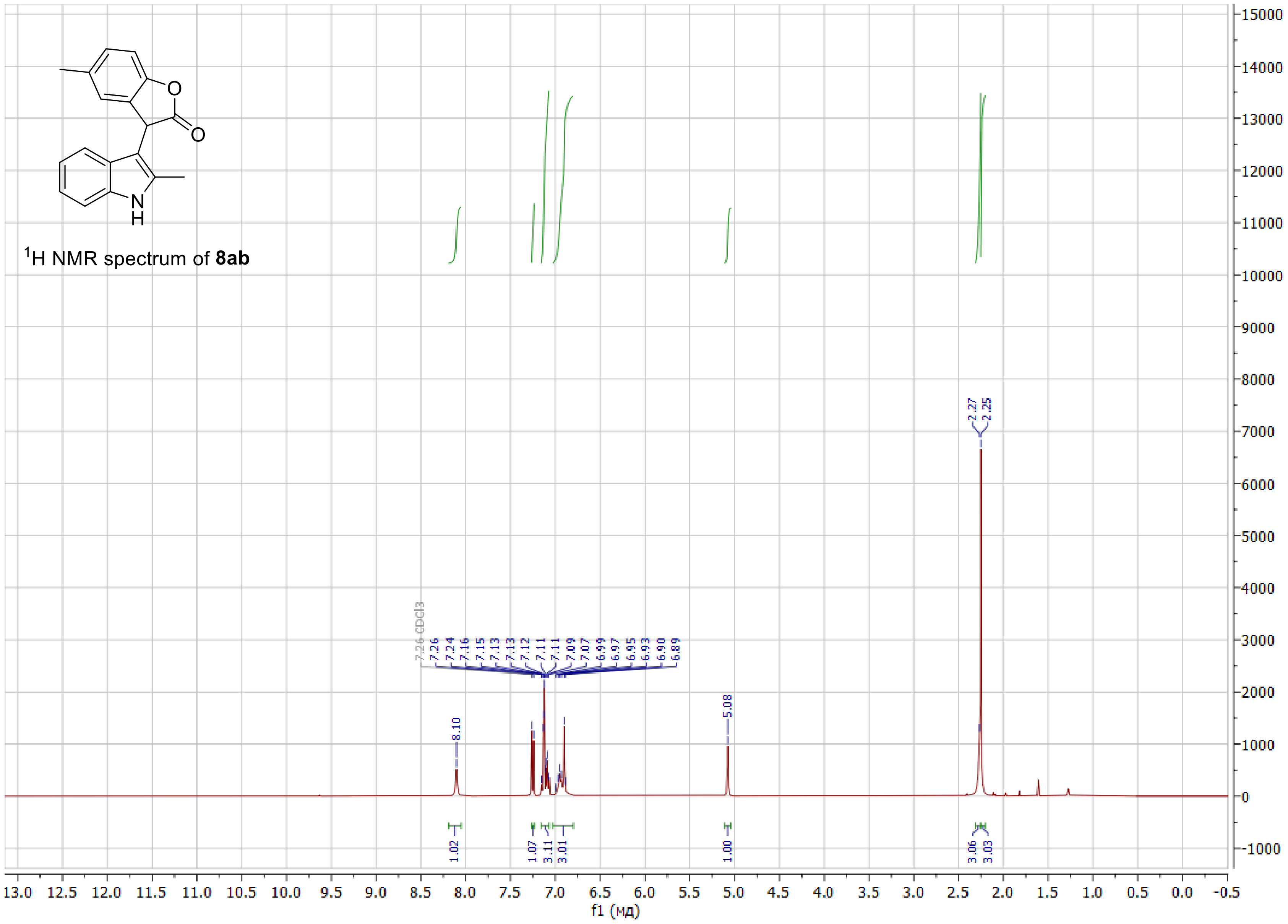

Figure S5. ^{1}H NMR spectrum of **8ab**

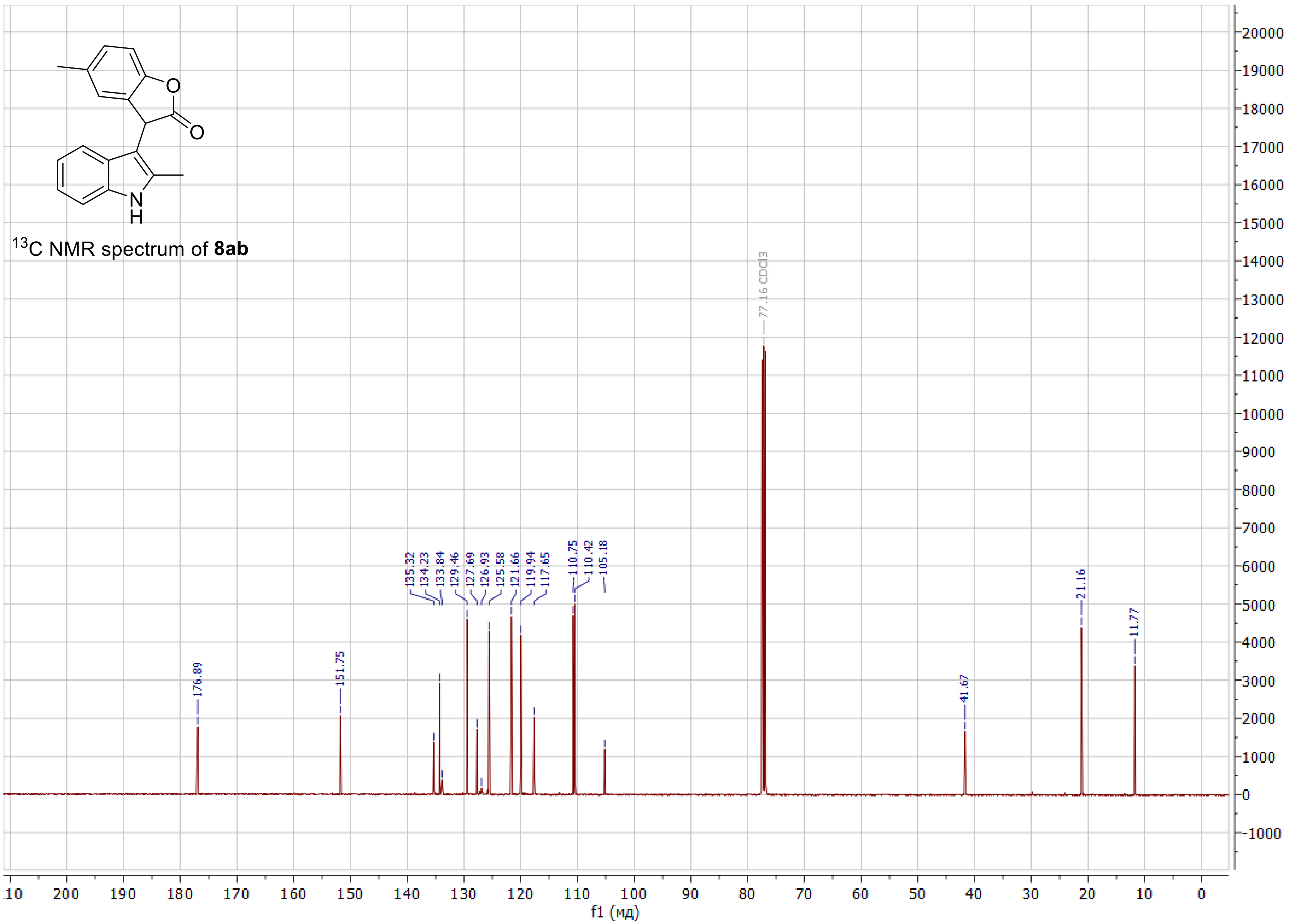

Figure S6. ^{13}C NMR spectrum of **8ab**

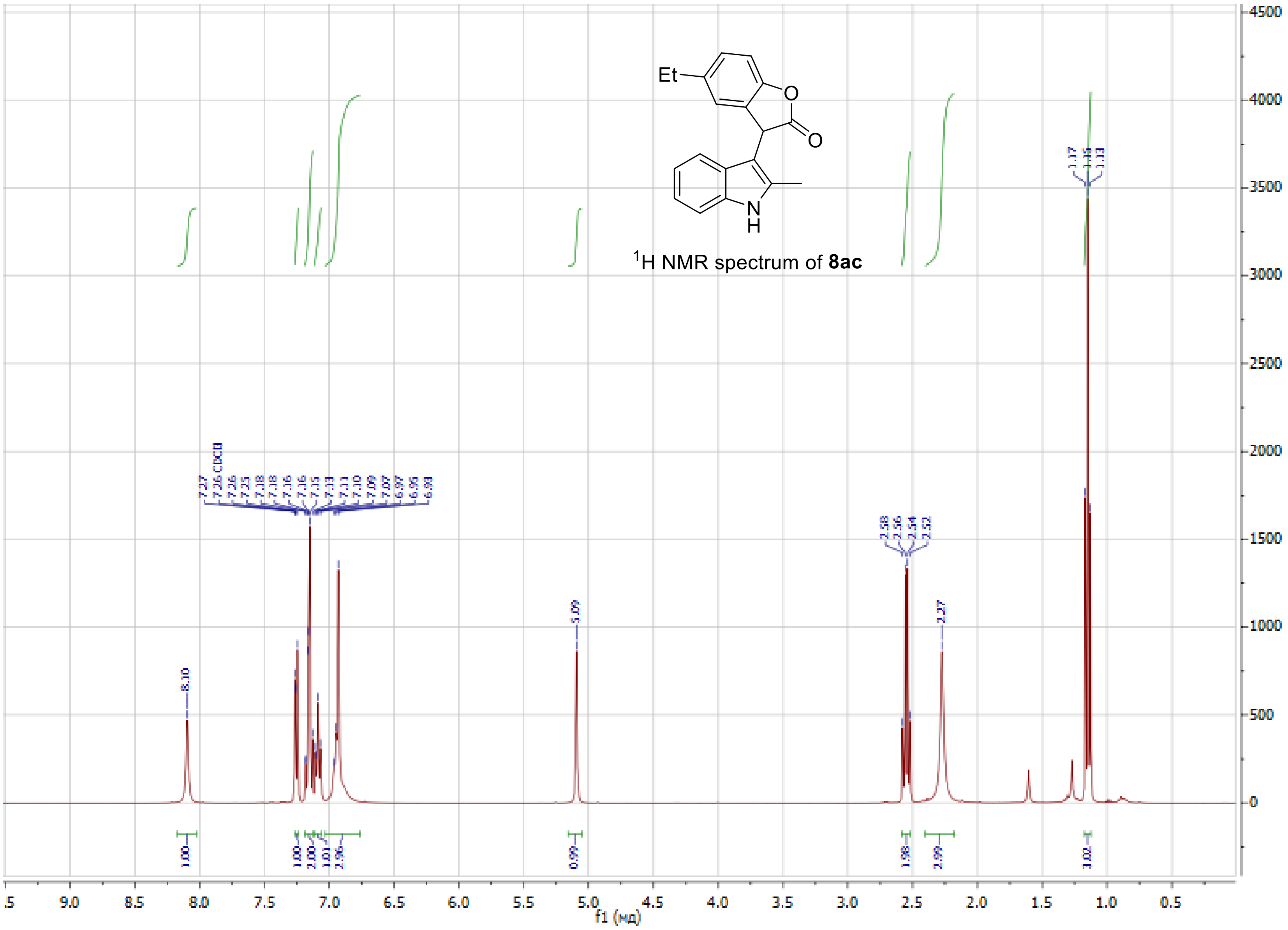

Figure S7. ^{1}H NMR spectrum of **8ac**

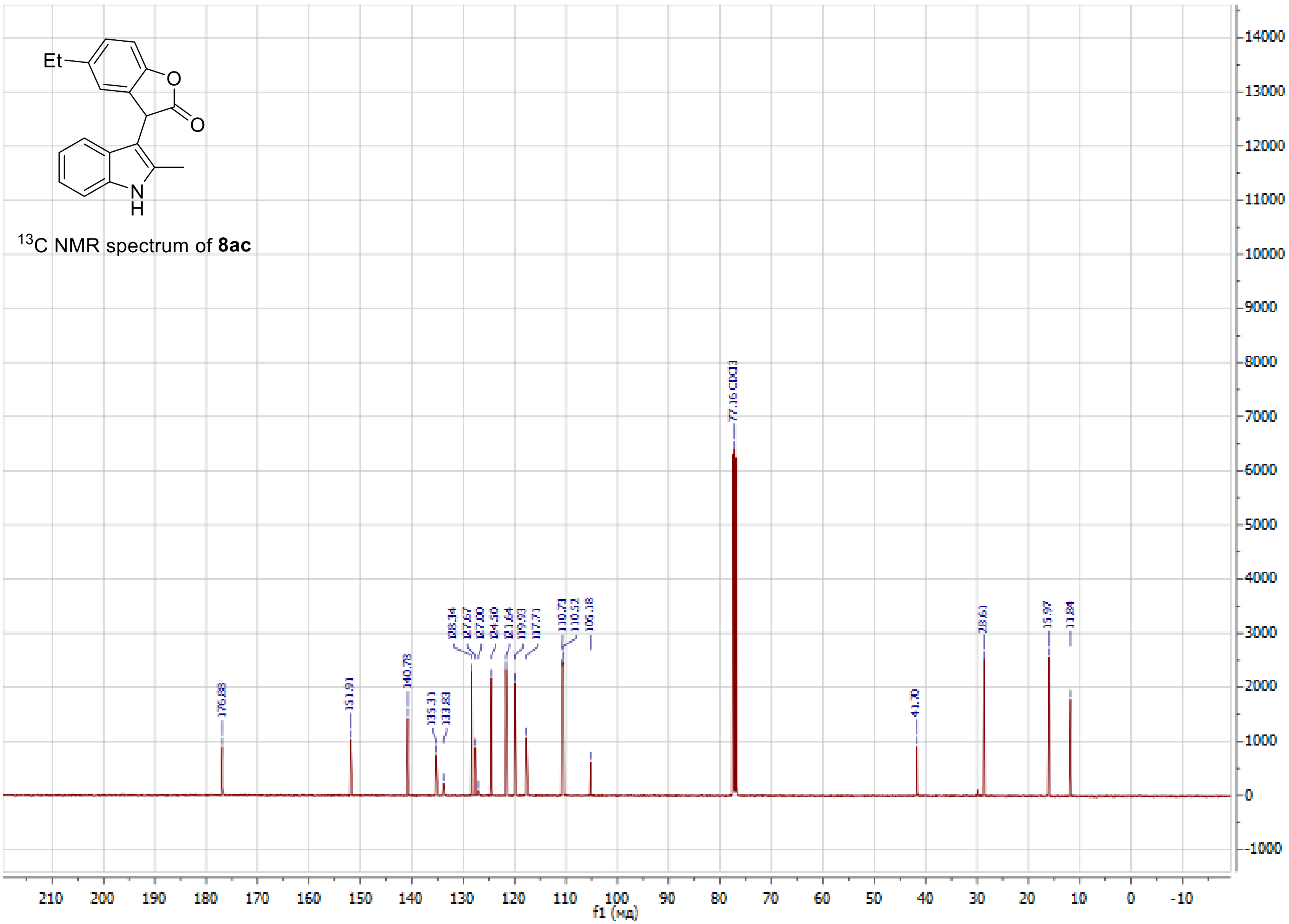

Figure S8. ^{13}C NMR spectrum of **8ac**

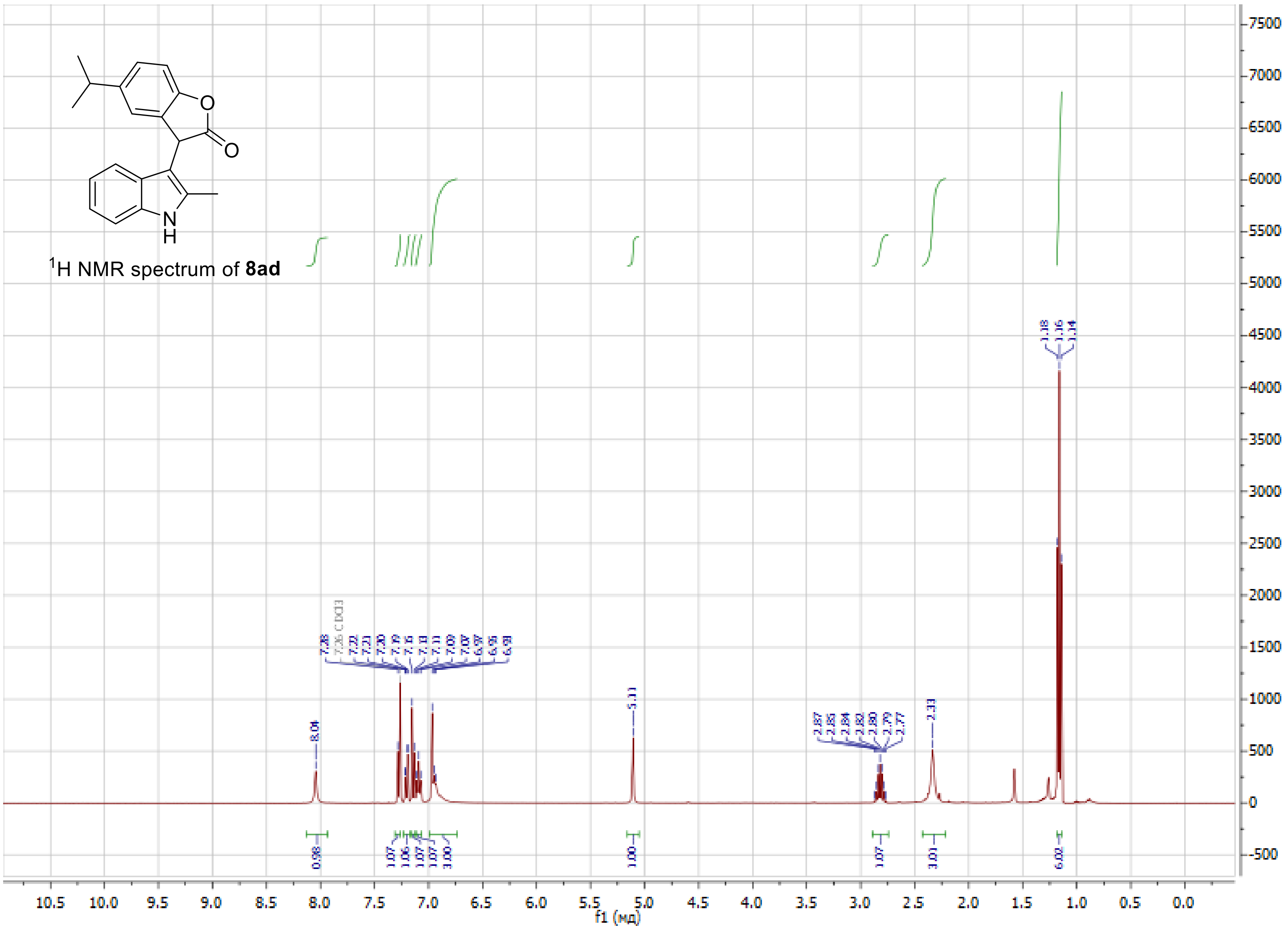

Figure S9. ^{1}H NMR spectrum of **8ad**

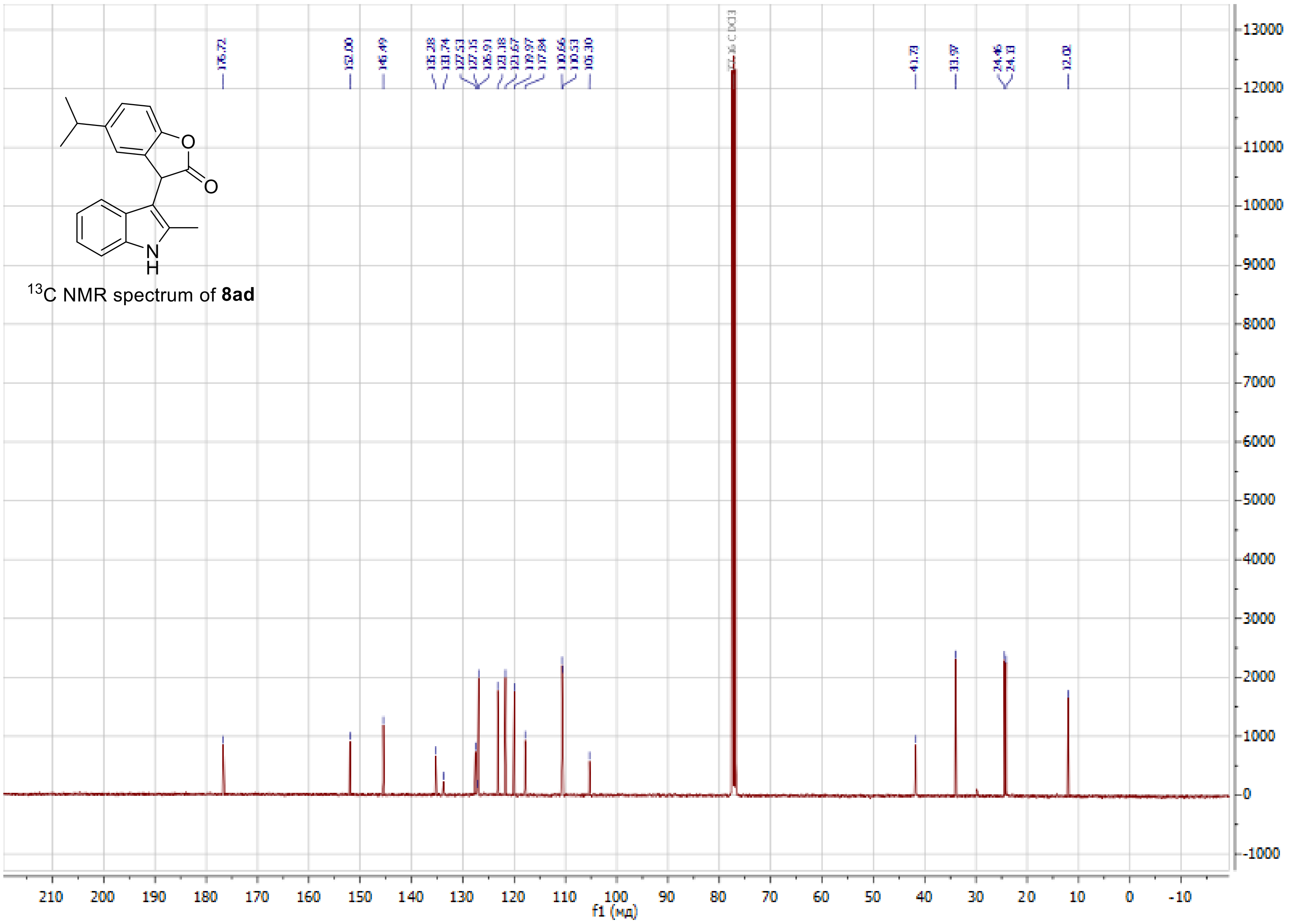

Figure S10. ^{13}C NMR spectrum of **8ad**

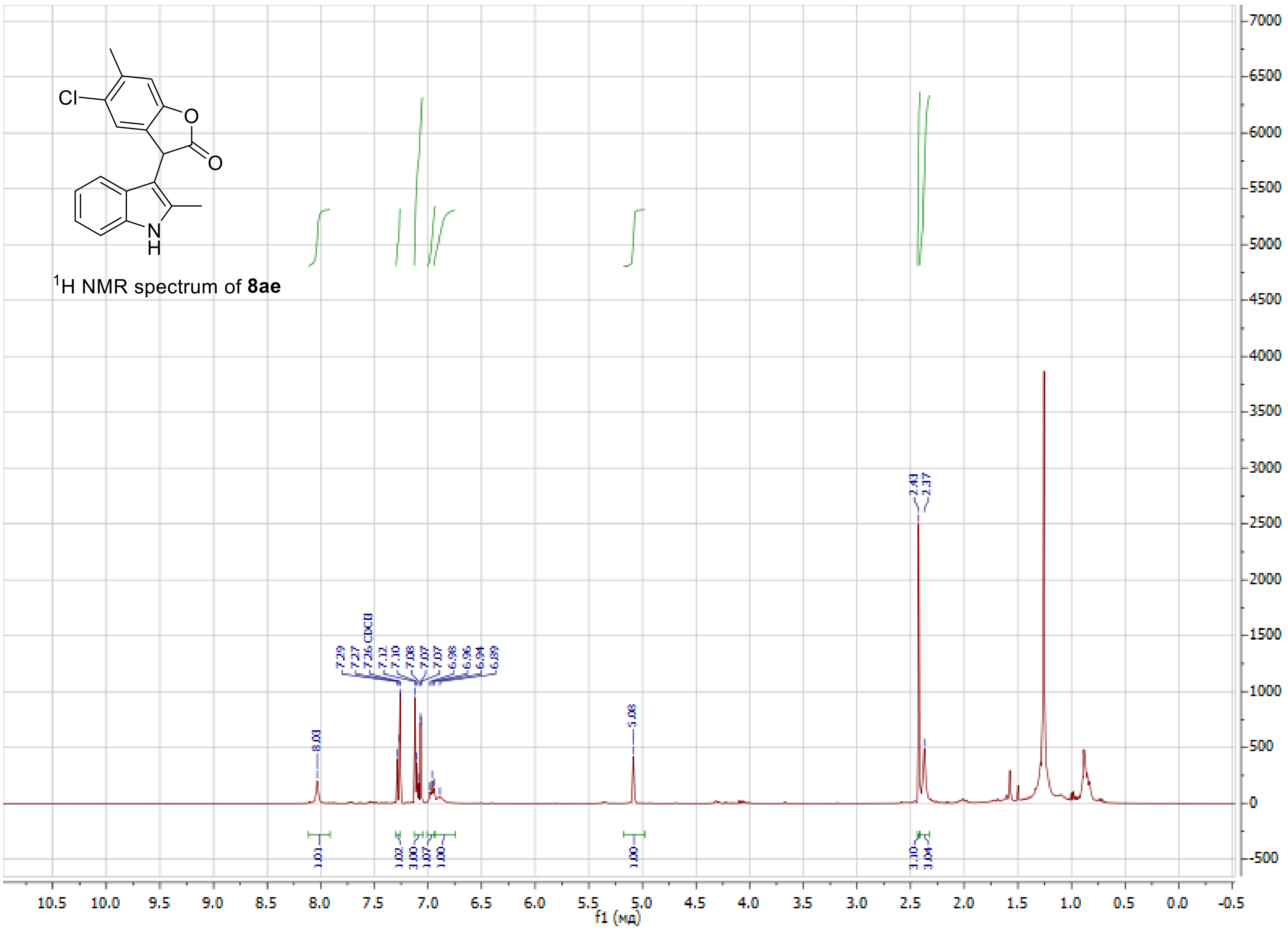

Figure S11. ^{1}H NMR spectrum of **8ae**

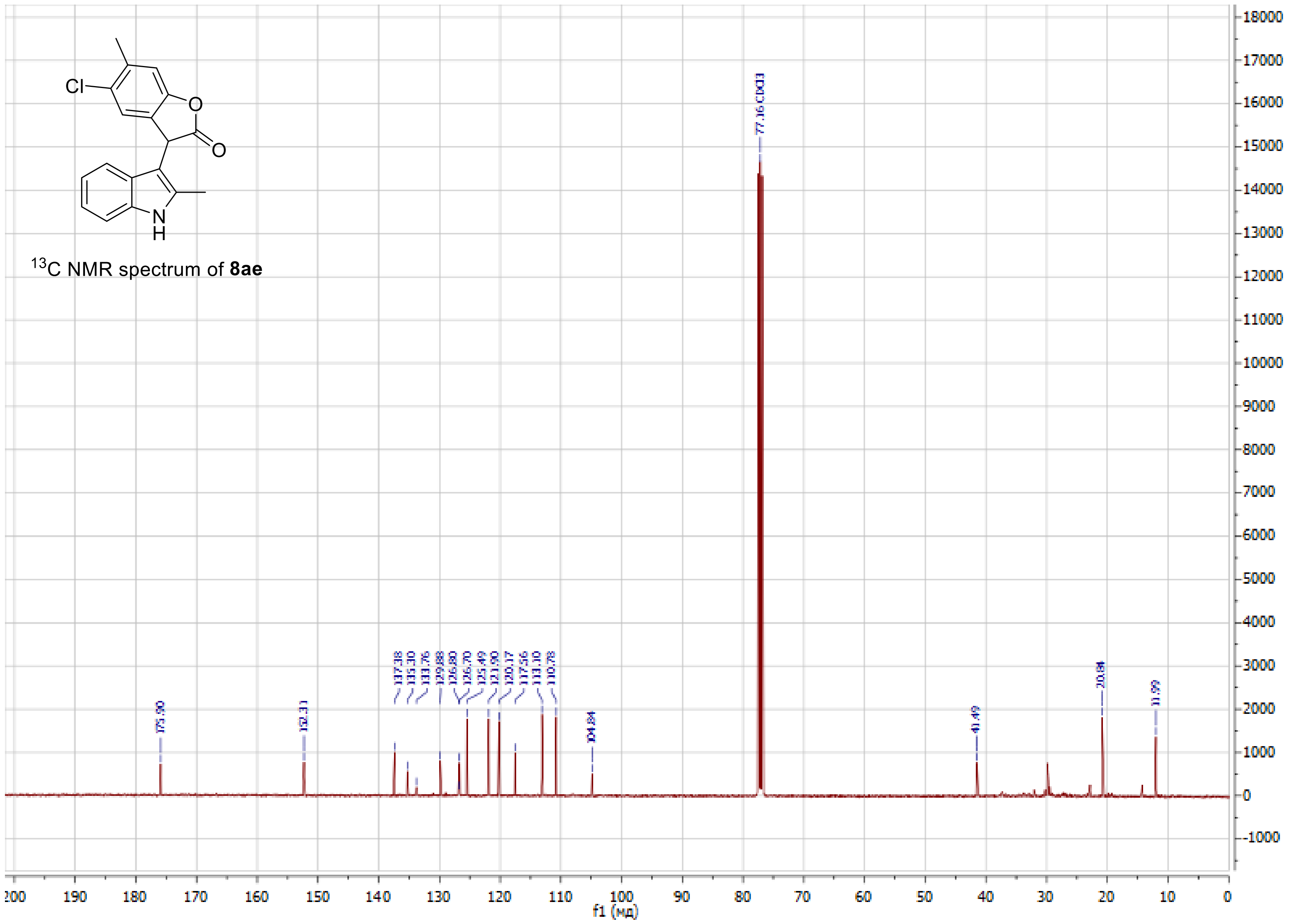

Figure S12. ^{13}C NMR spectrum of **8ae**

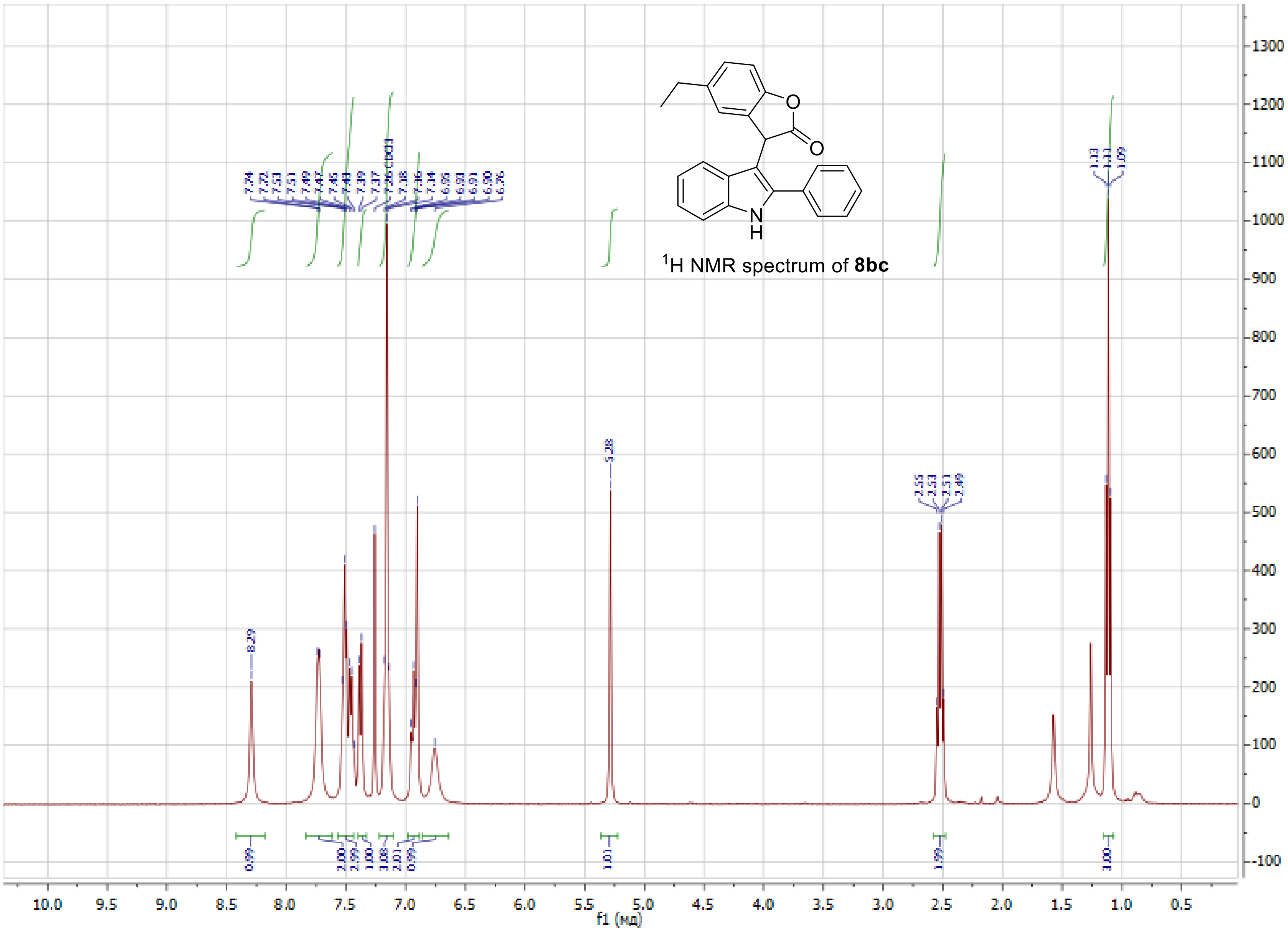

Figure S13. ^{1}H NMR spectrum of **8bc**

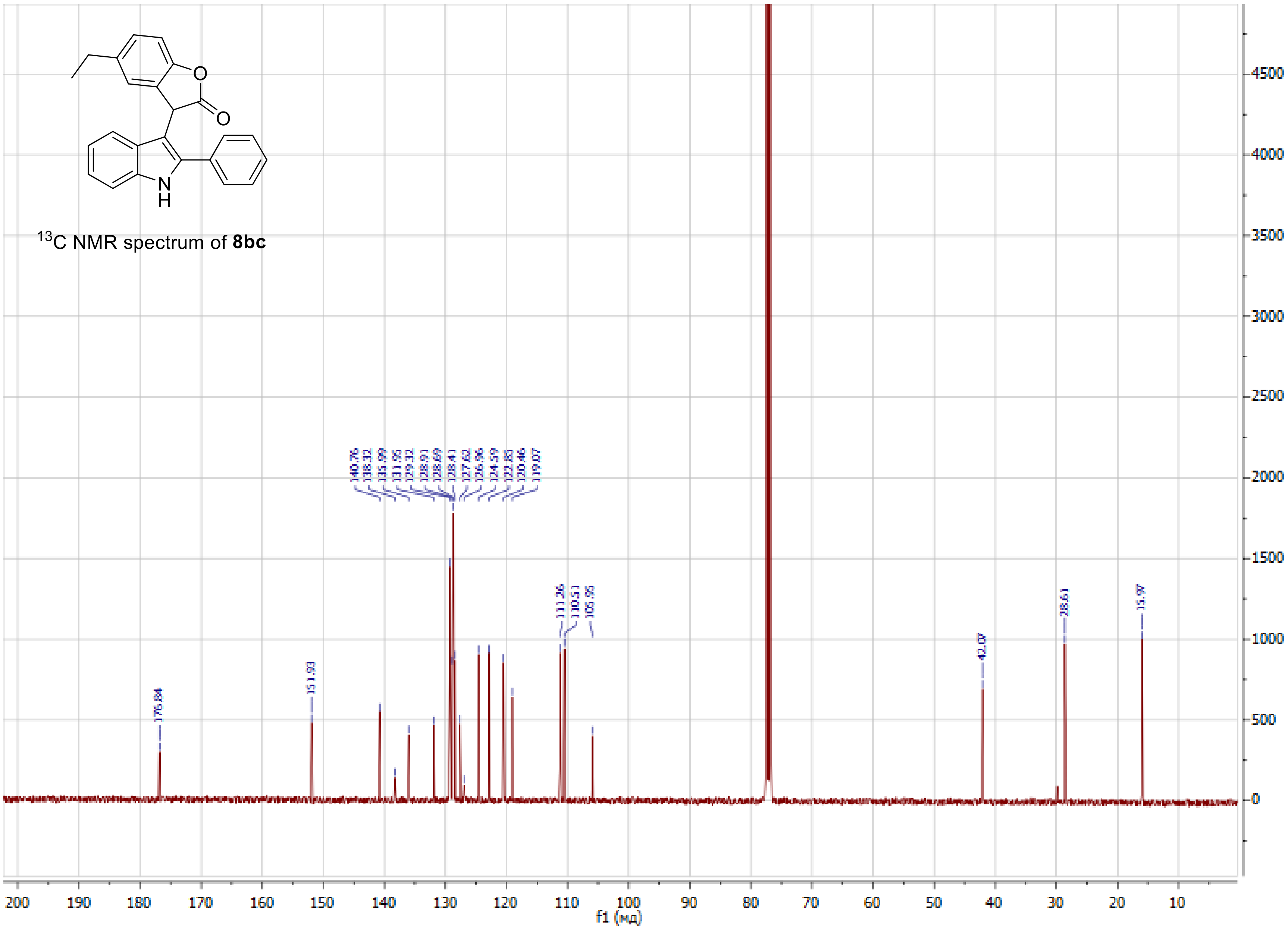

Figure S14. ^{13}C NMR spectrum of **8bc**

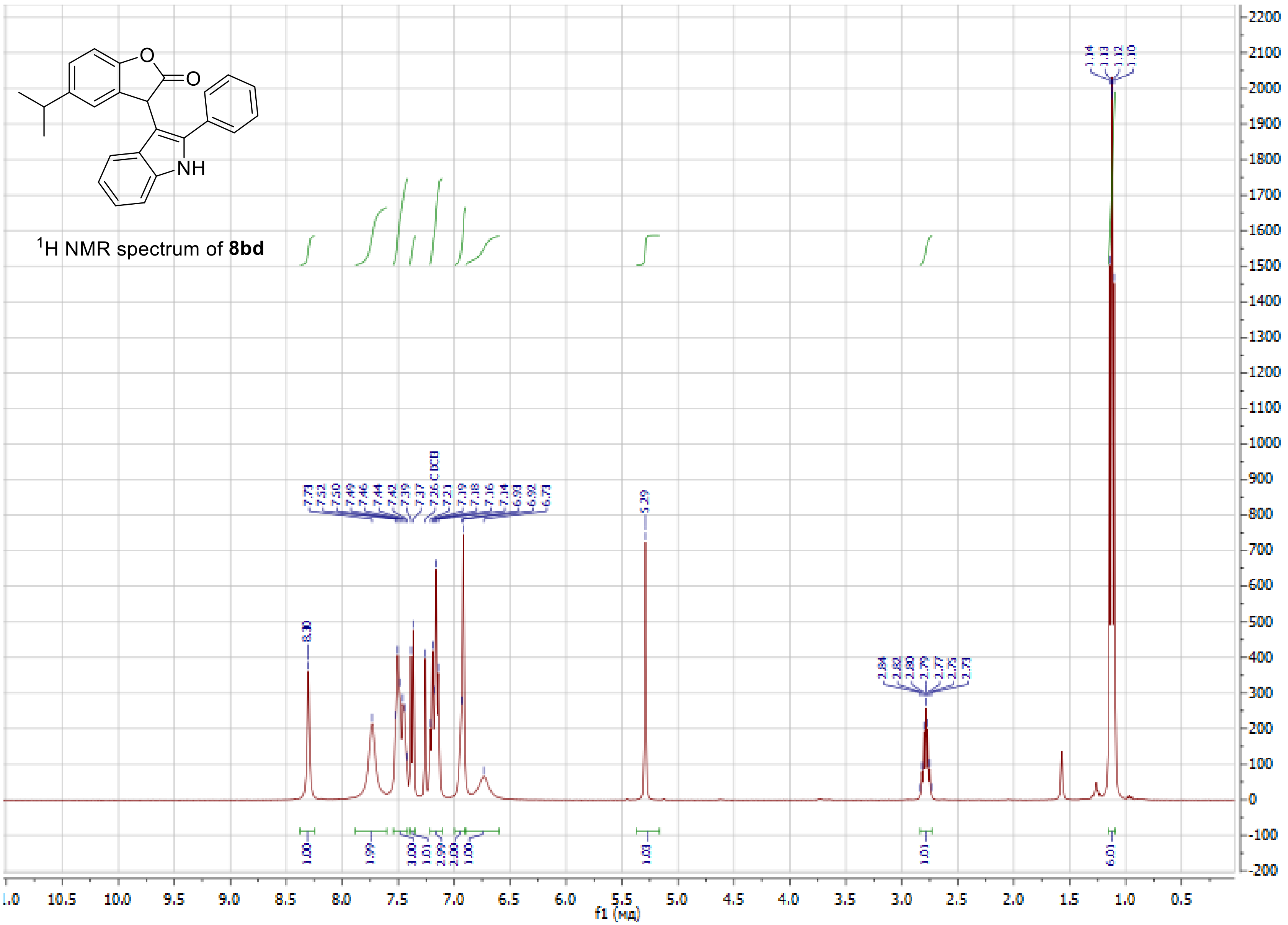

Figure S15. ^{1}H NMR spectrum of **8bd**

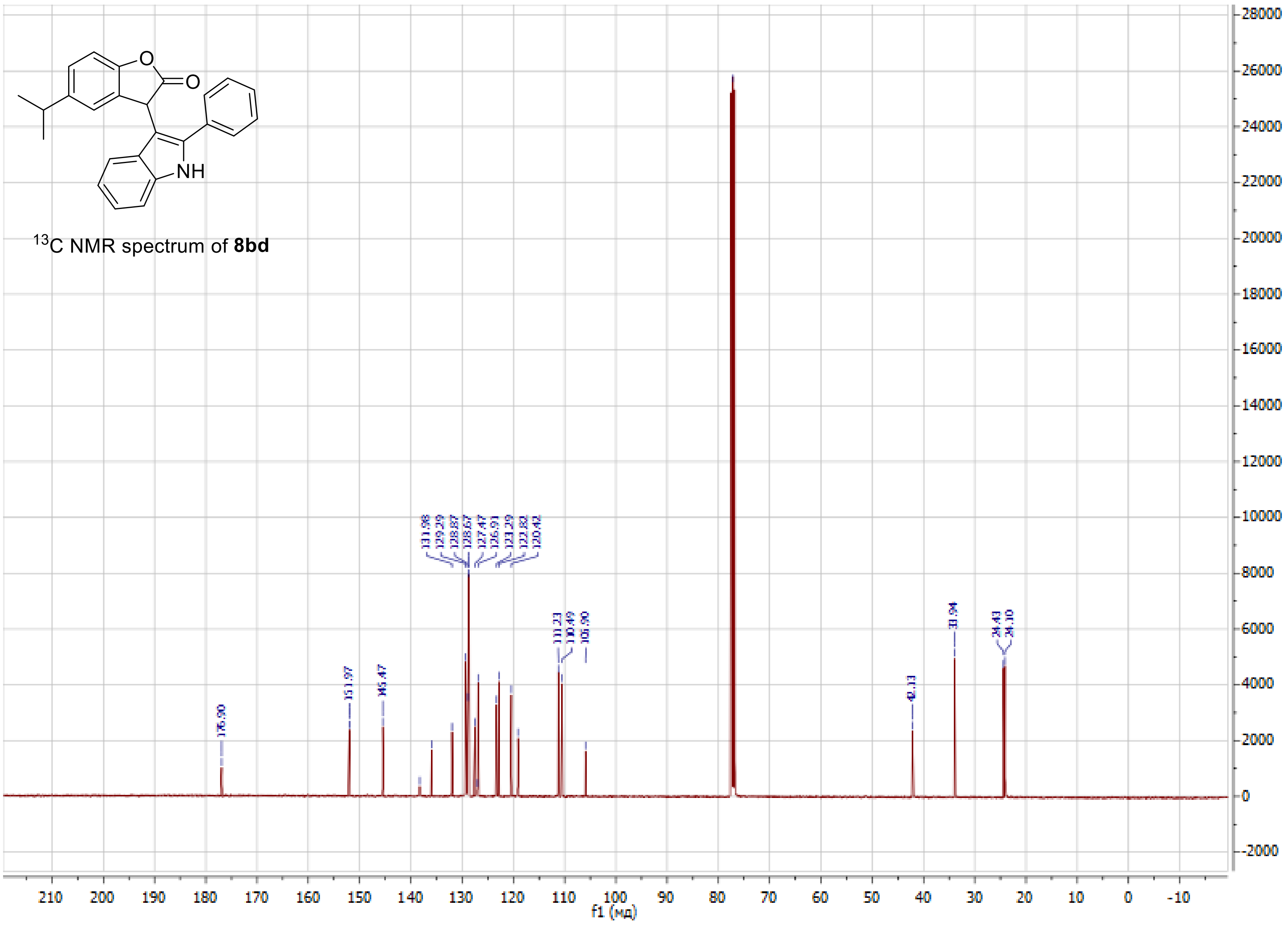

Figure S16. ^{13}C NMR spectrum of **8bd**

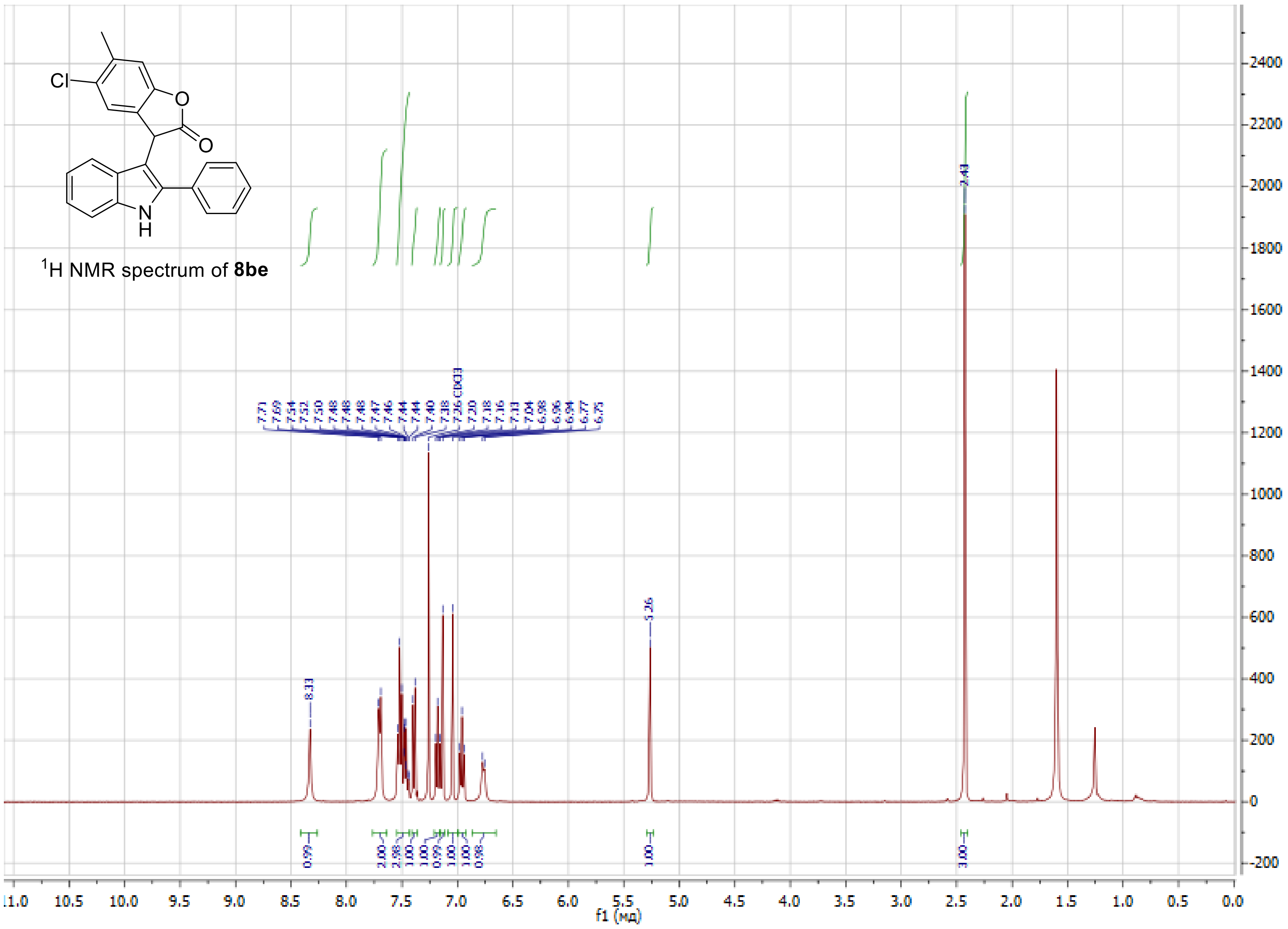

Figure S17. ^{1}H NMR spectrum of **8be**

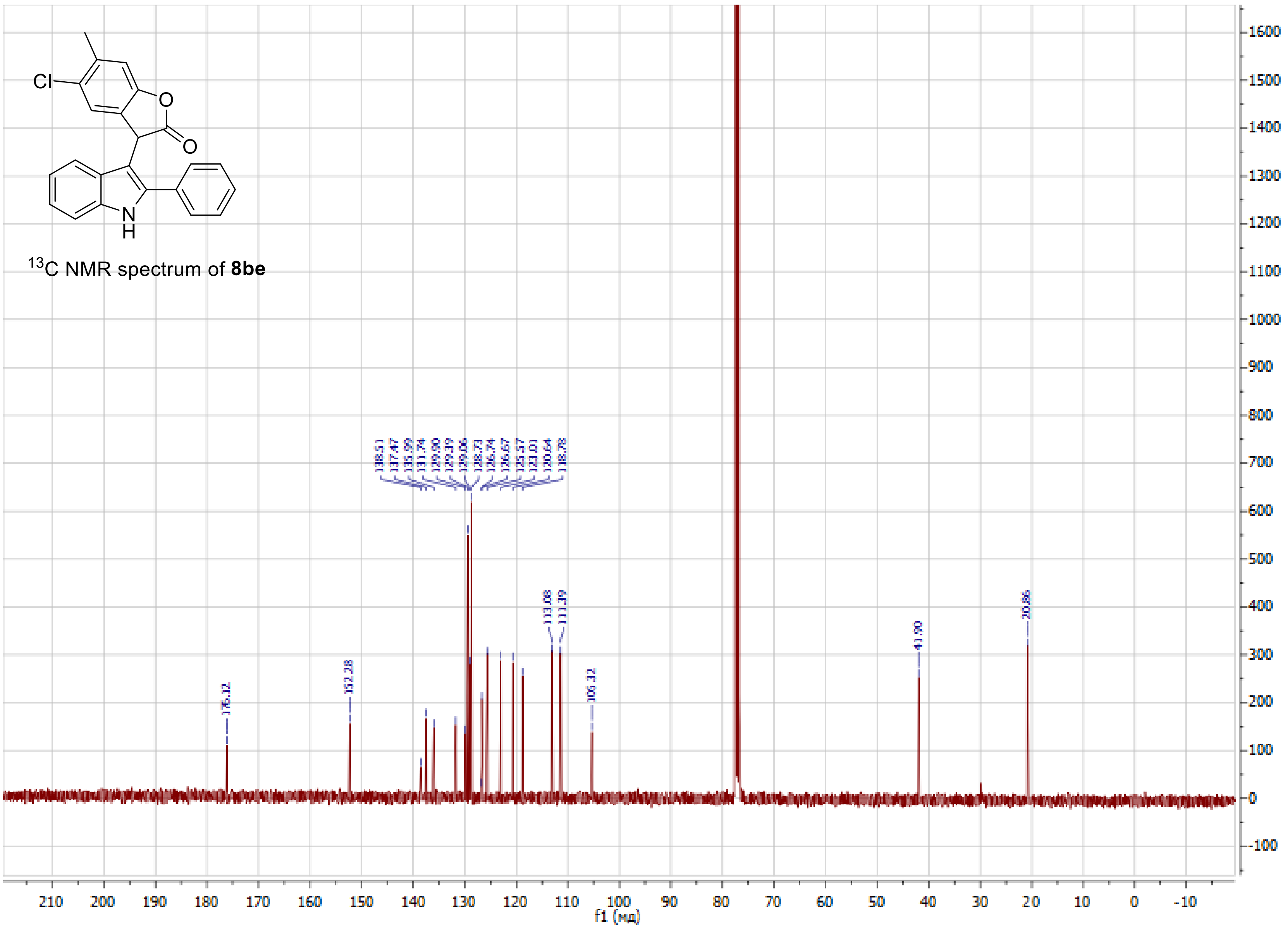

Figure S18. ^{13}C NMR spectrum of **8be**

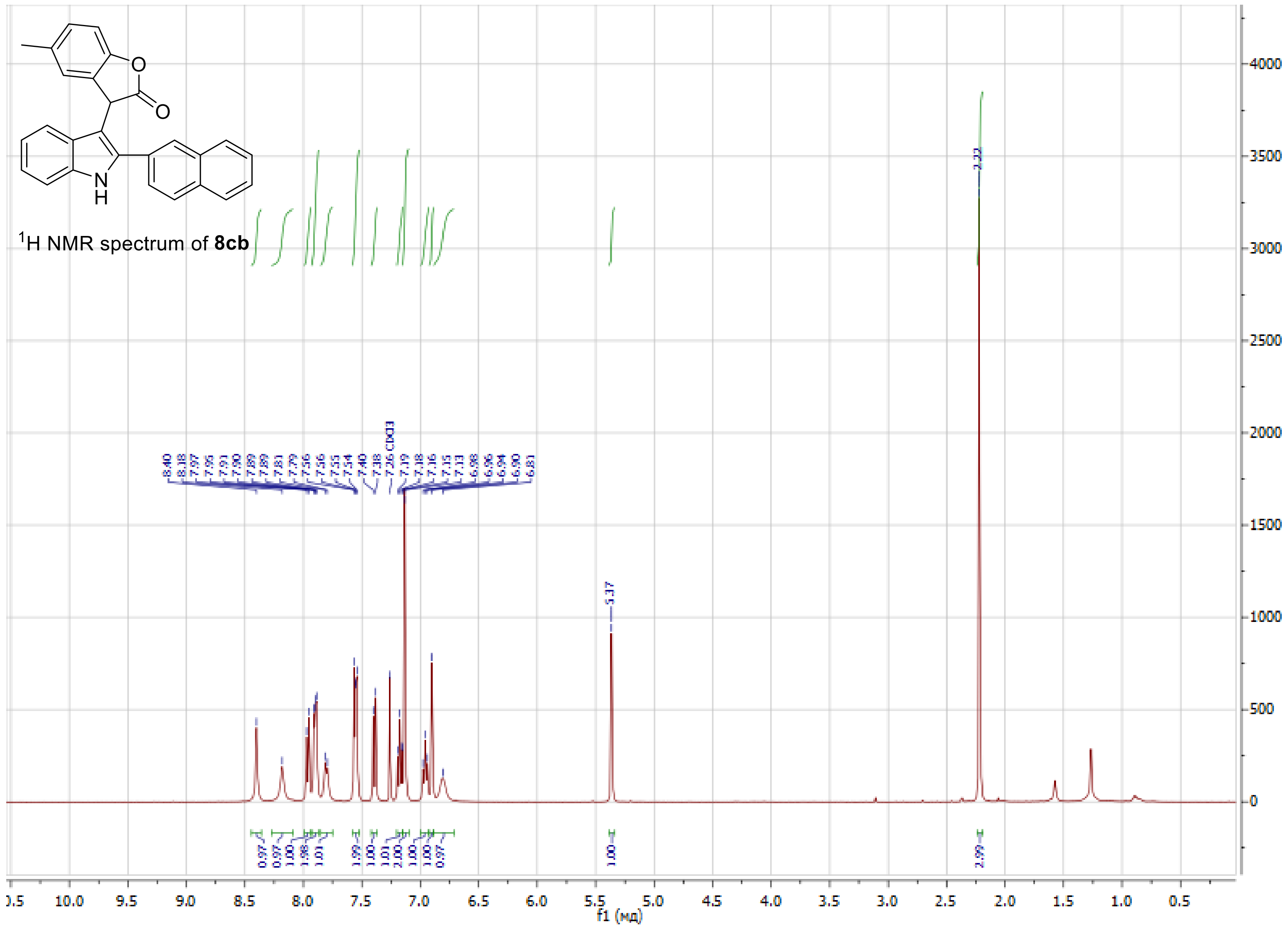

Figure S19. ^{1}H NMR spectrum of **8cb**

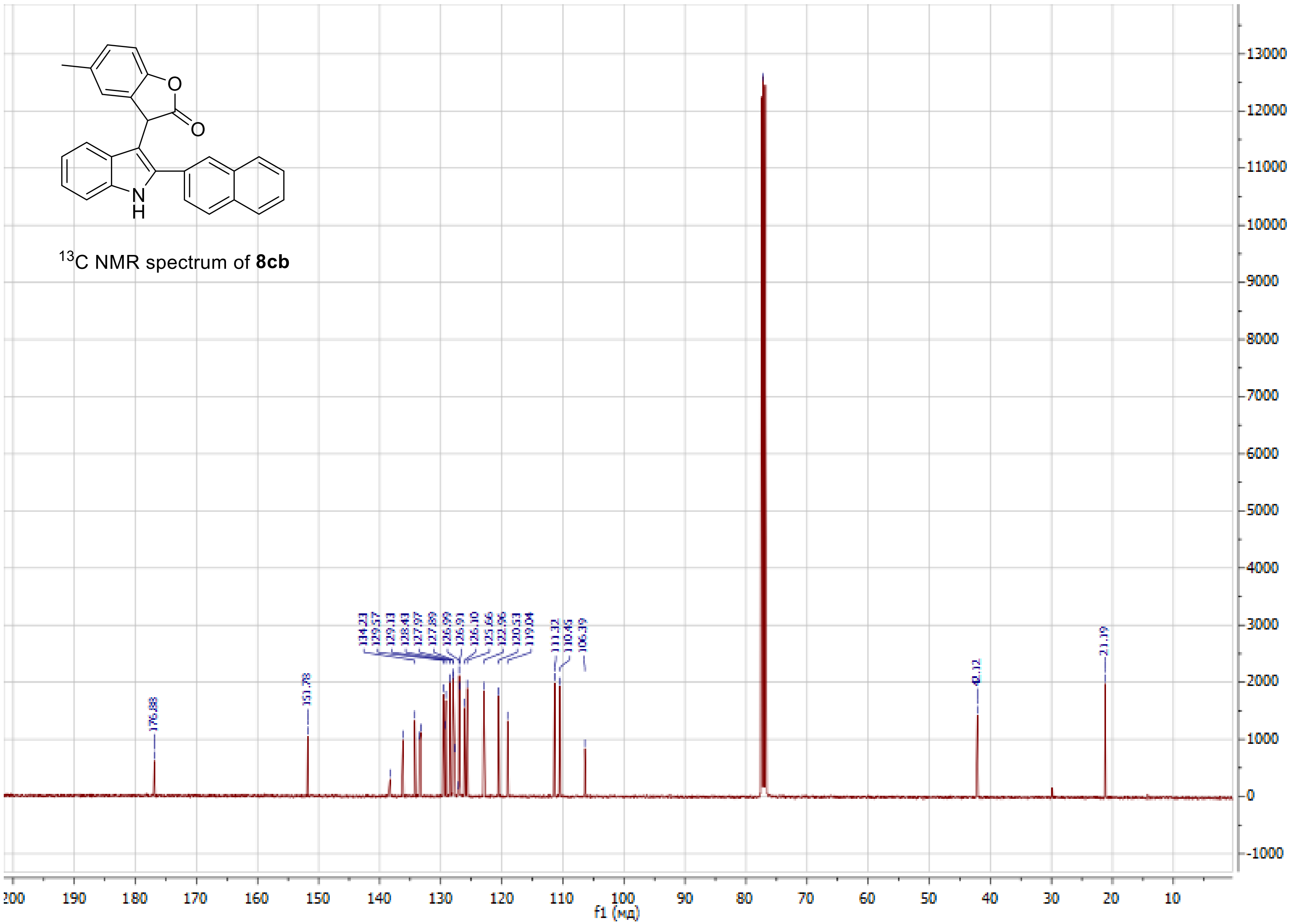

Figure S20. ^{13}C NMR spectrum of **8cb**

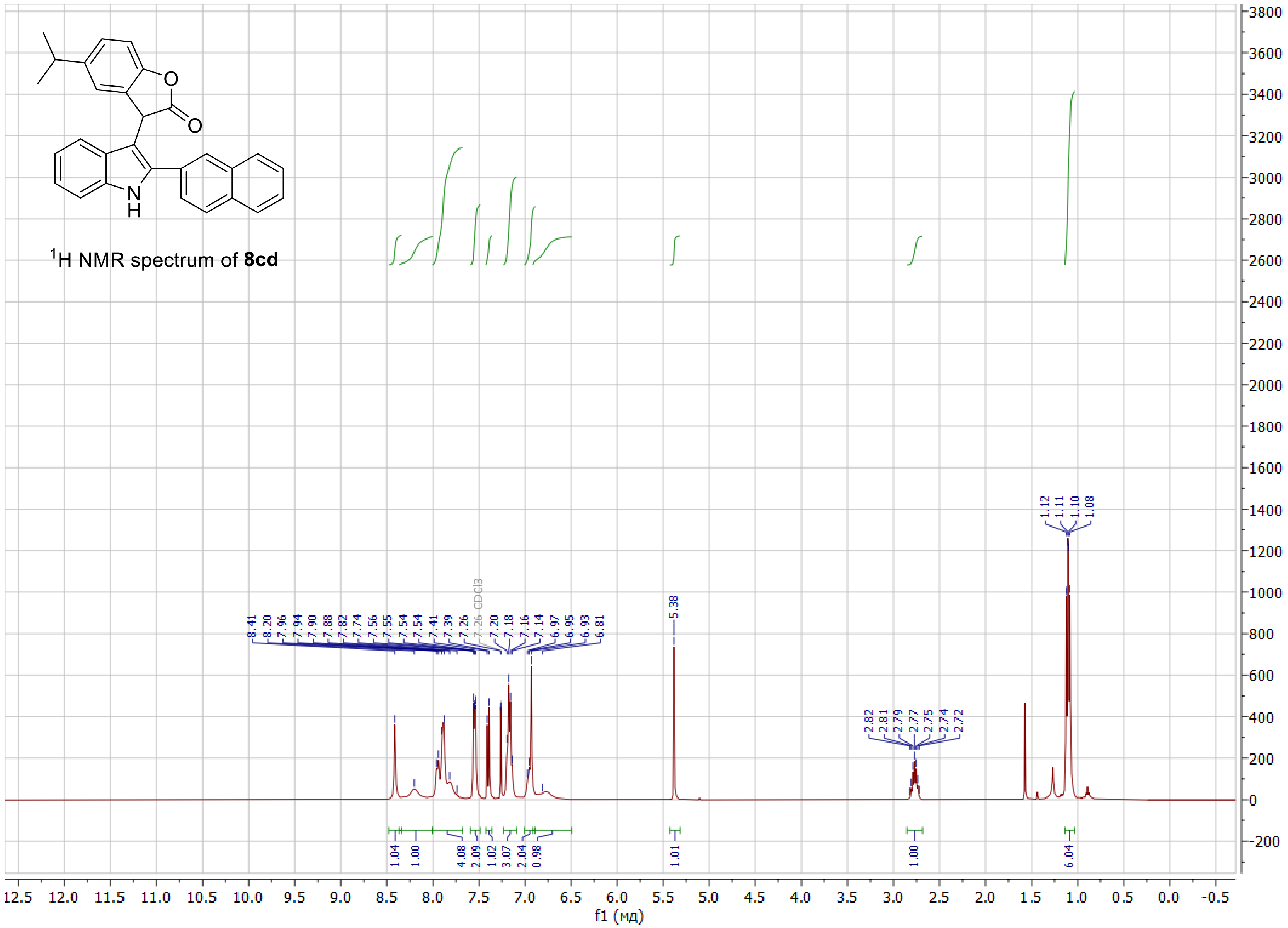

Figure S21. ^{1}H NMR spectrum of **8cd**

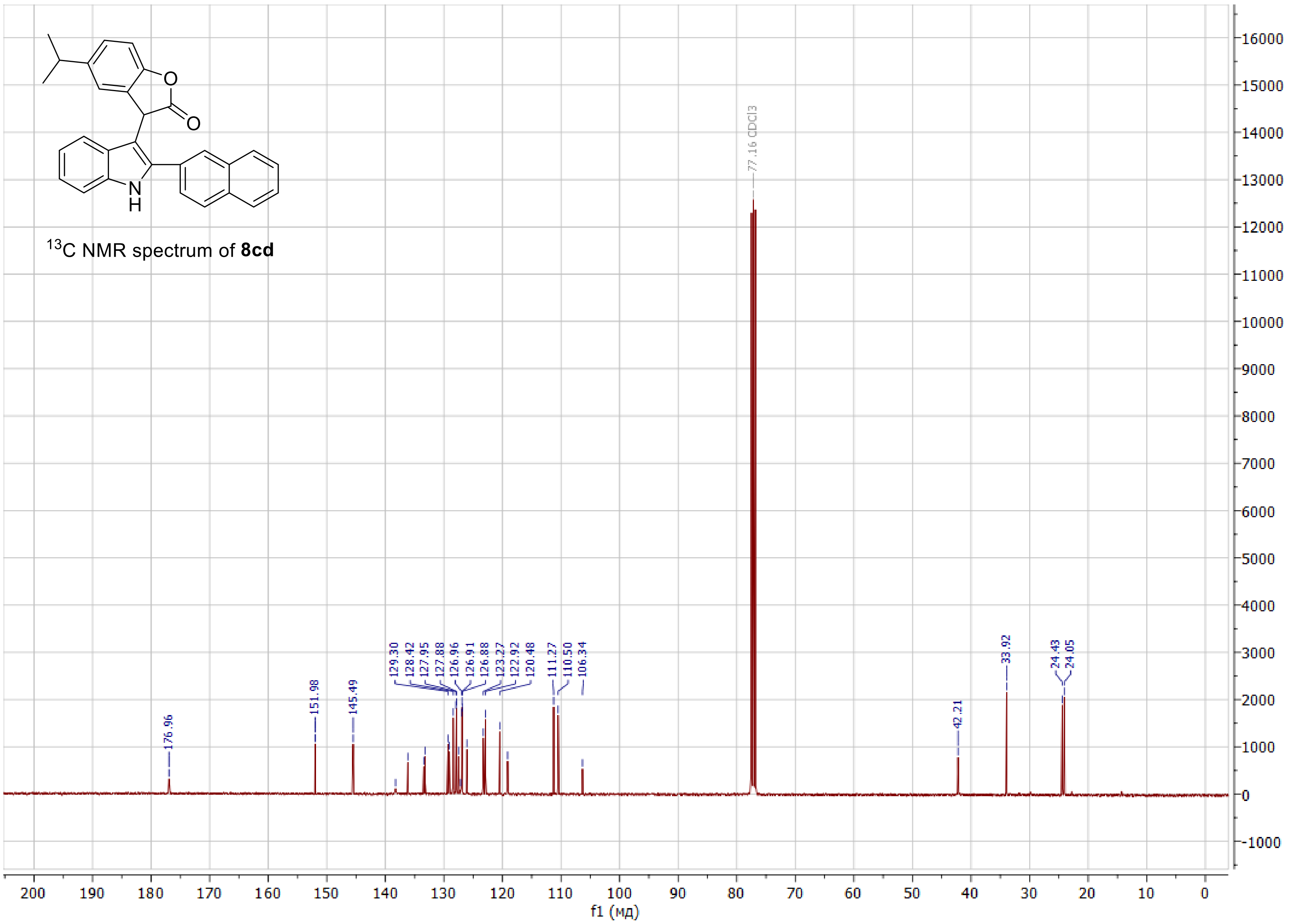

Figure S22. ^{13}C NMR spectrum of **8cd**

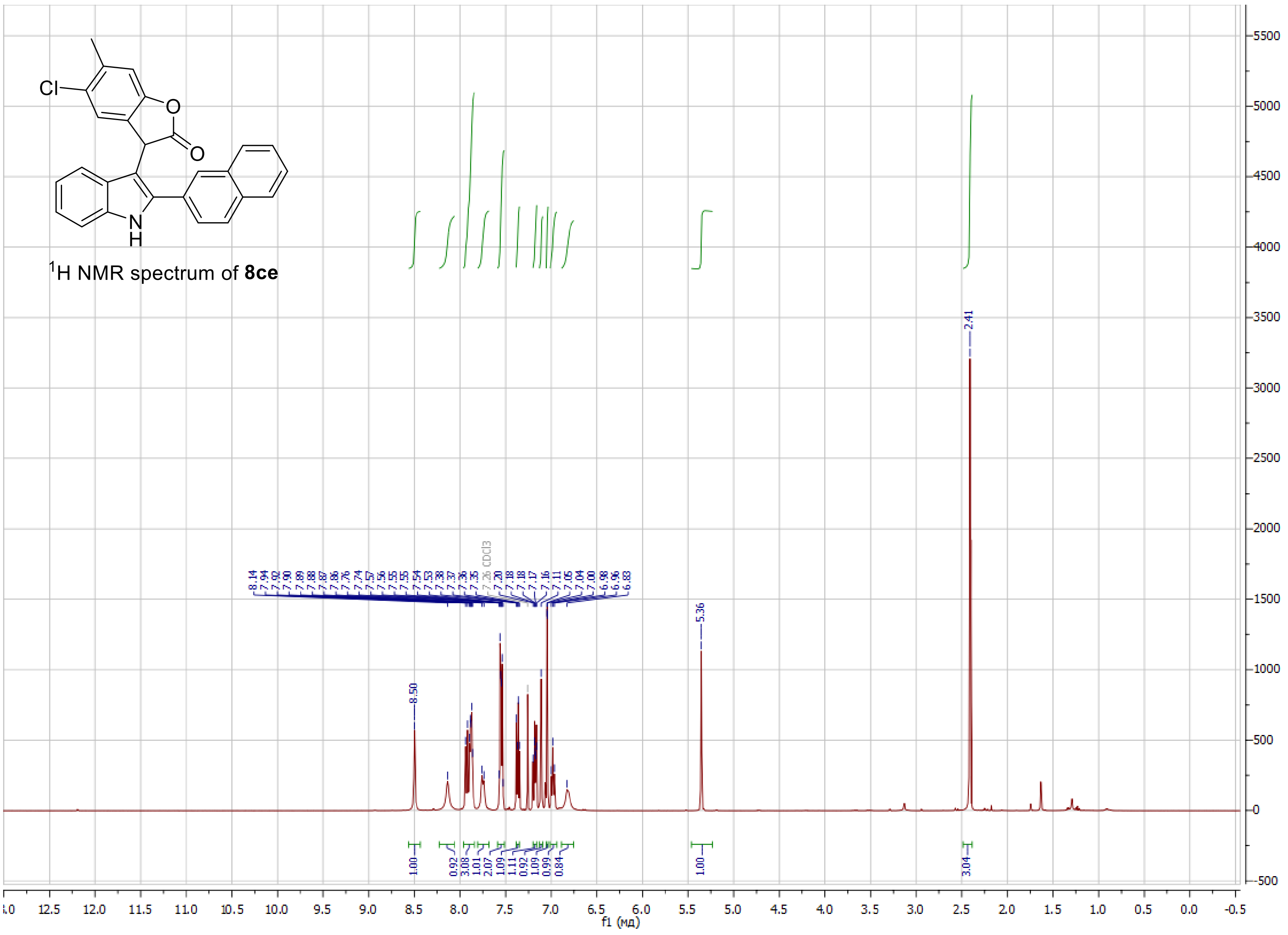

Figure S23. ^{1}H NMR spectrum of **8ce**

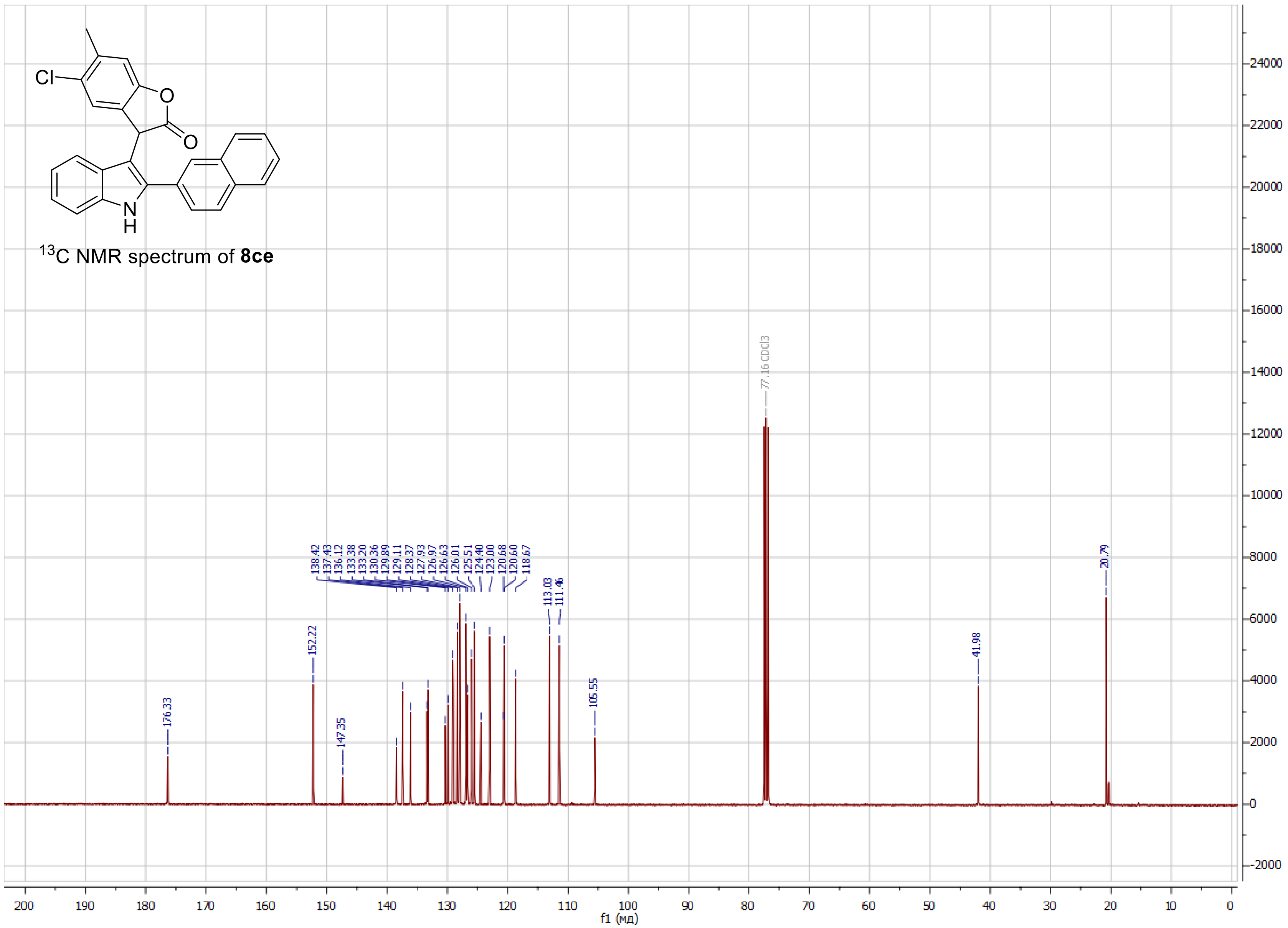

Figure S24. ^{13}C NMR spectrum of **8ce**

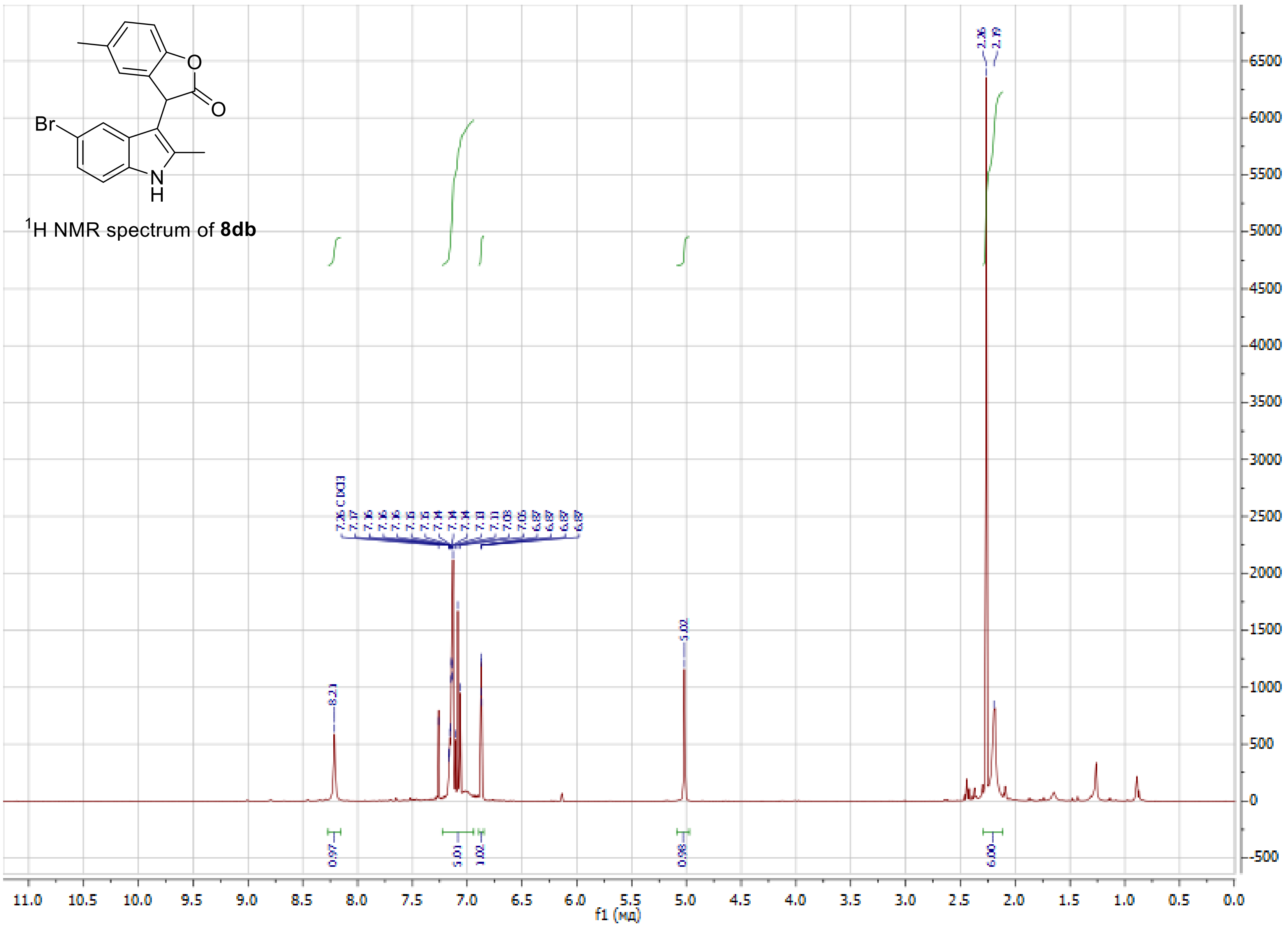

Figure S25. ^{1}H NMR spectrum of **8db**

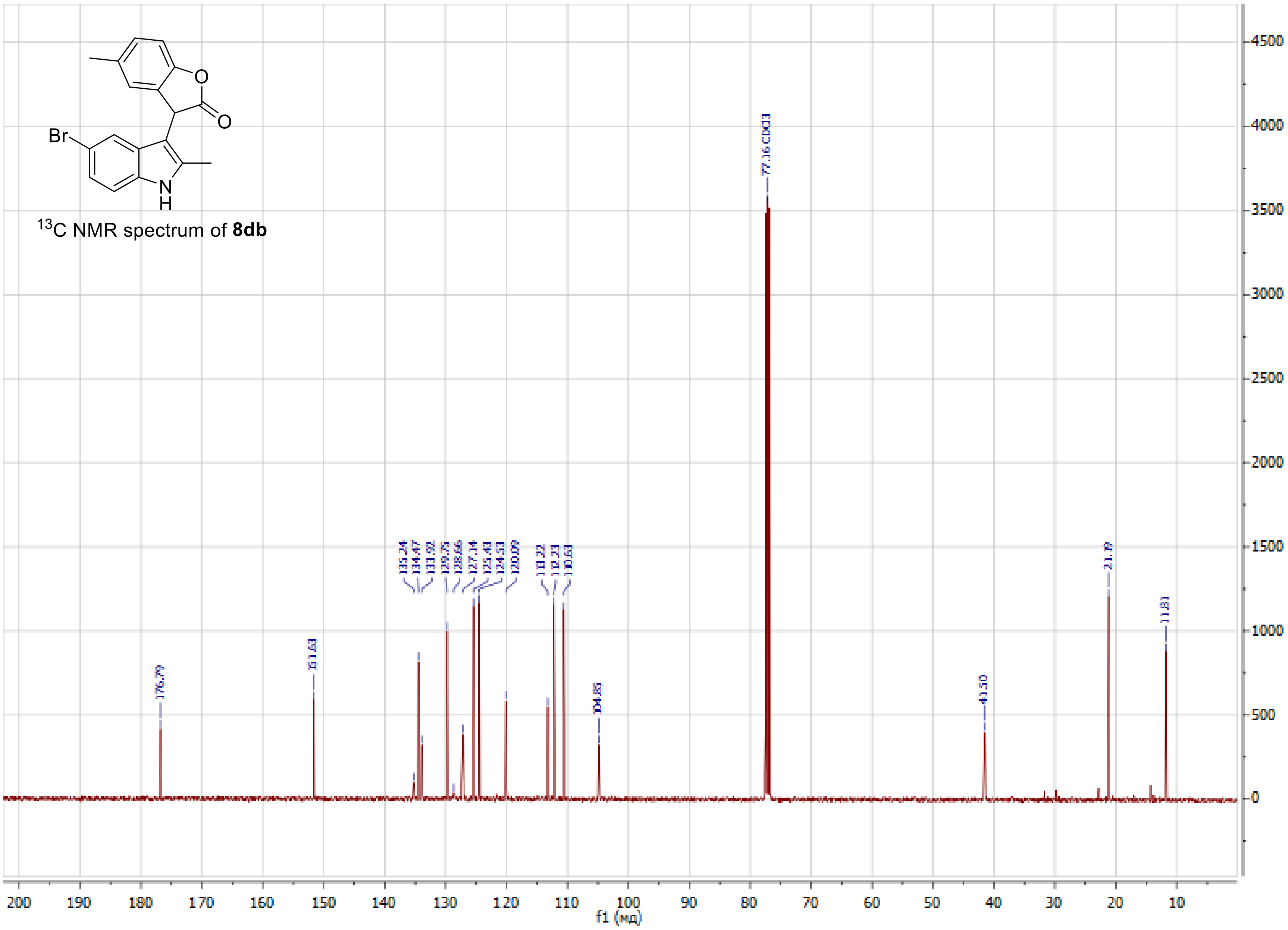

Figure S26. ^{13}C NMR spectrum of **8db**

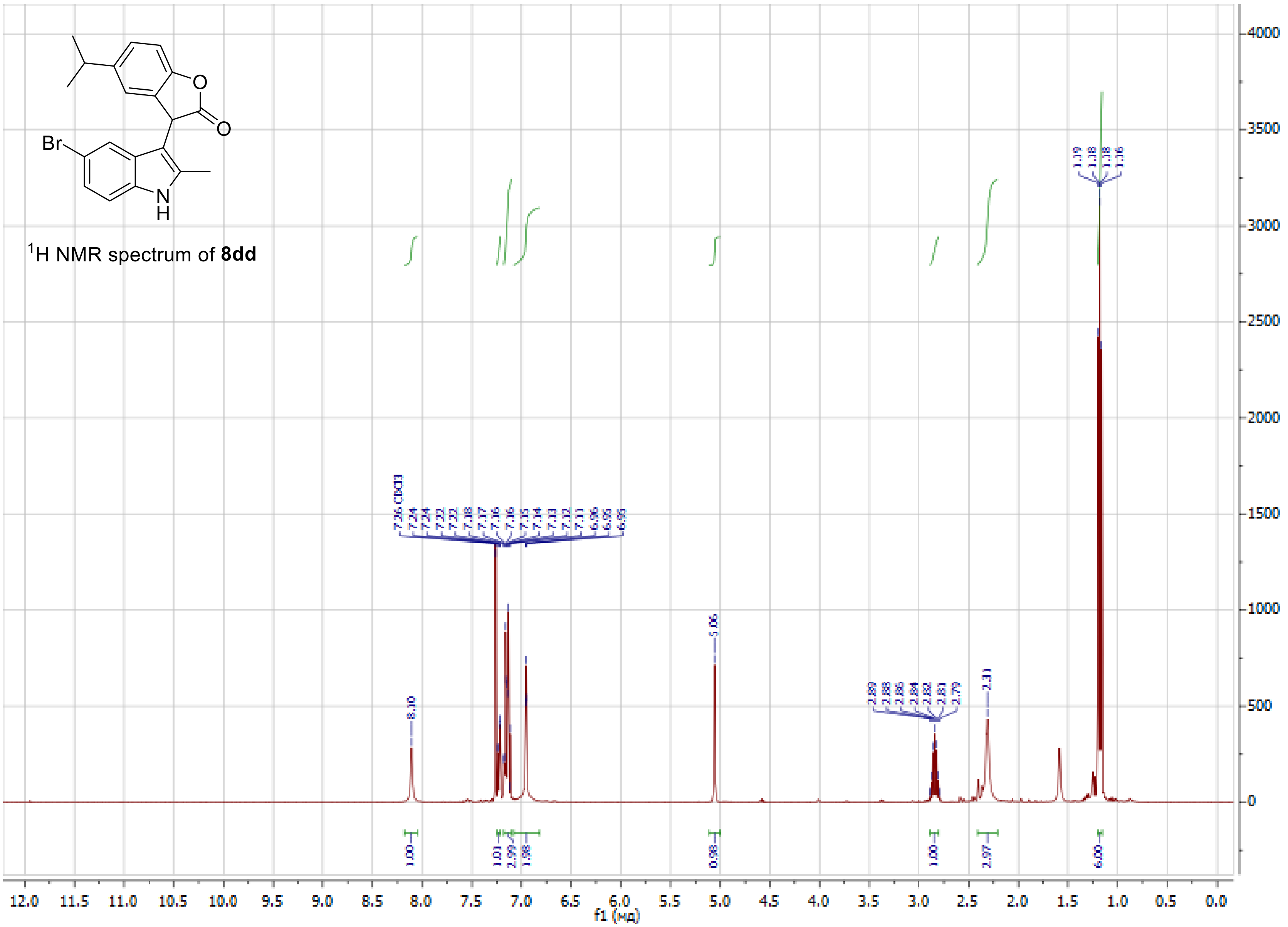

Figure S27. ^{1}H NMR spectrum of **8dd**

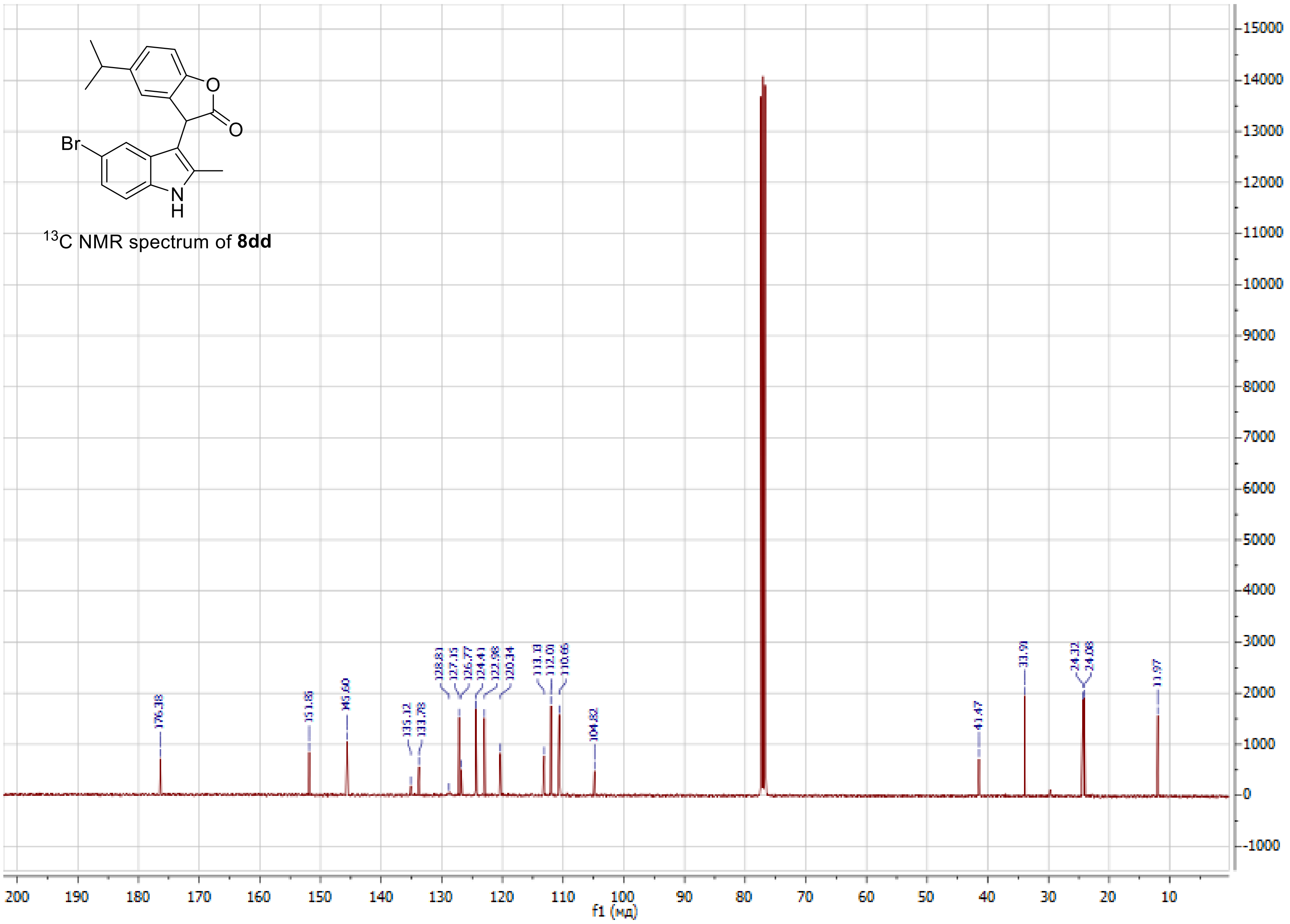

Figure S28. ^{13}C NMR spectrum of **8dd**

^{1}H and ^{13}C NMR spectral charts for 4,5-Dimethyl-2-(1-(2-methyl-1*H*-indol-3-yl)-2-nitroethyl)phenol (9aa):

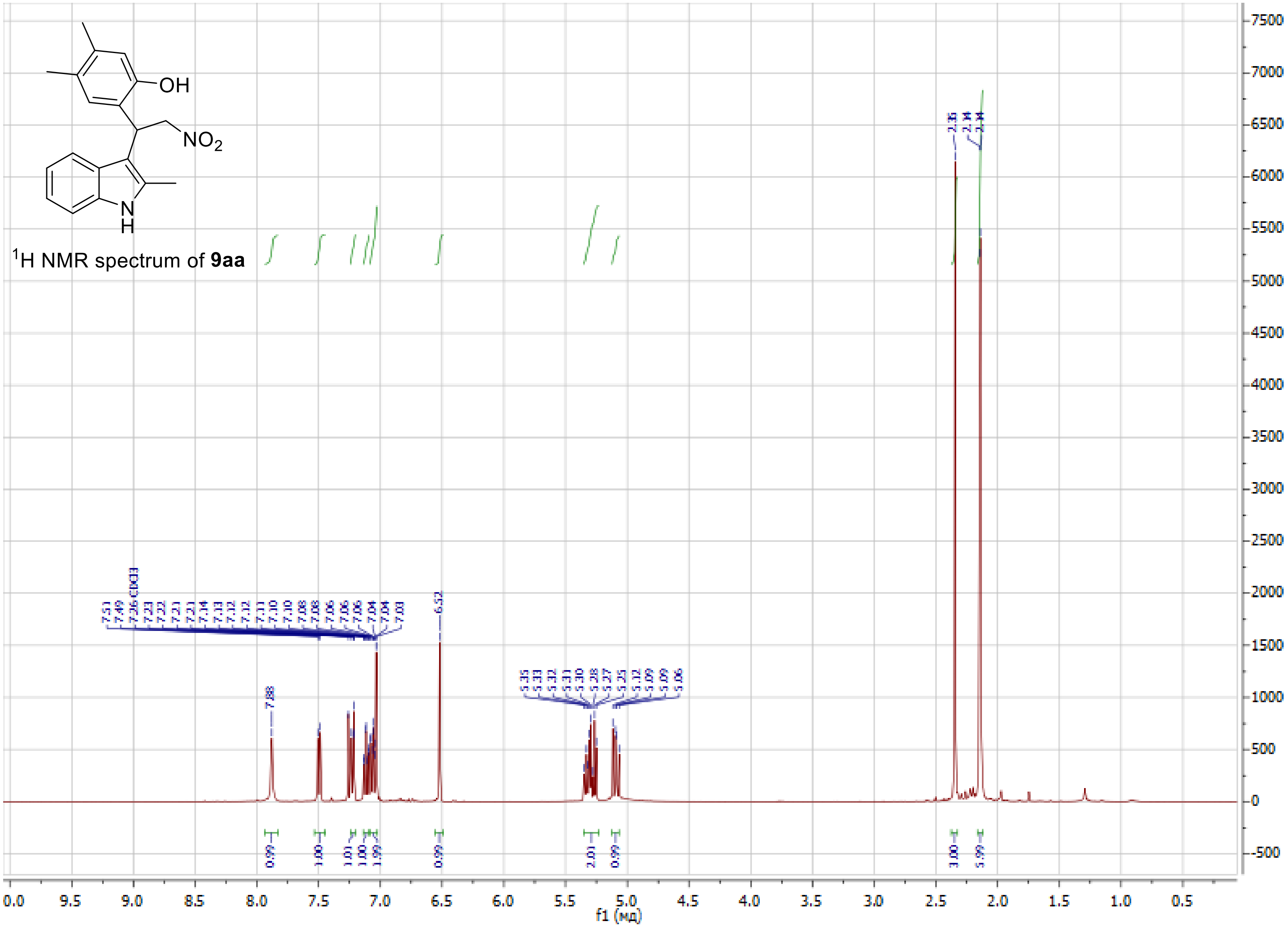

Figure S29. ^{1}H NMR spectrum of **9aa**

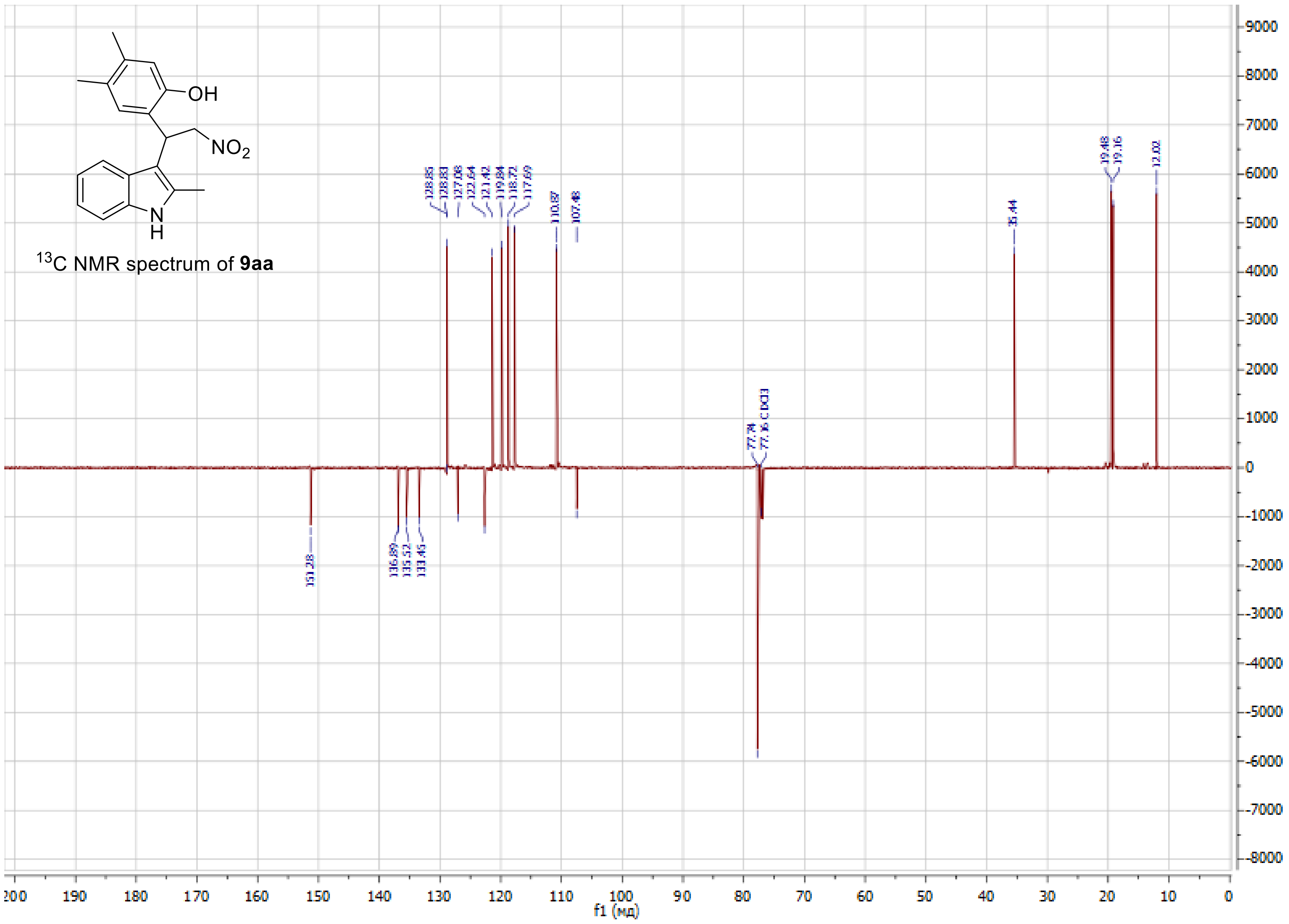

Figure S30. ^{13}C NMR spectrum of **9aa**

HRMS spectral charts

HRMS spectral chart for starting (E)-5-bromo-2-methyl-3-(2-nitrovinyl)-1H-indole (3d)

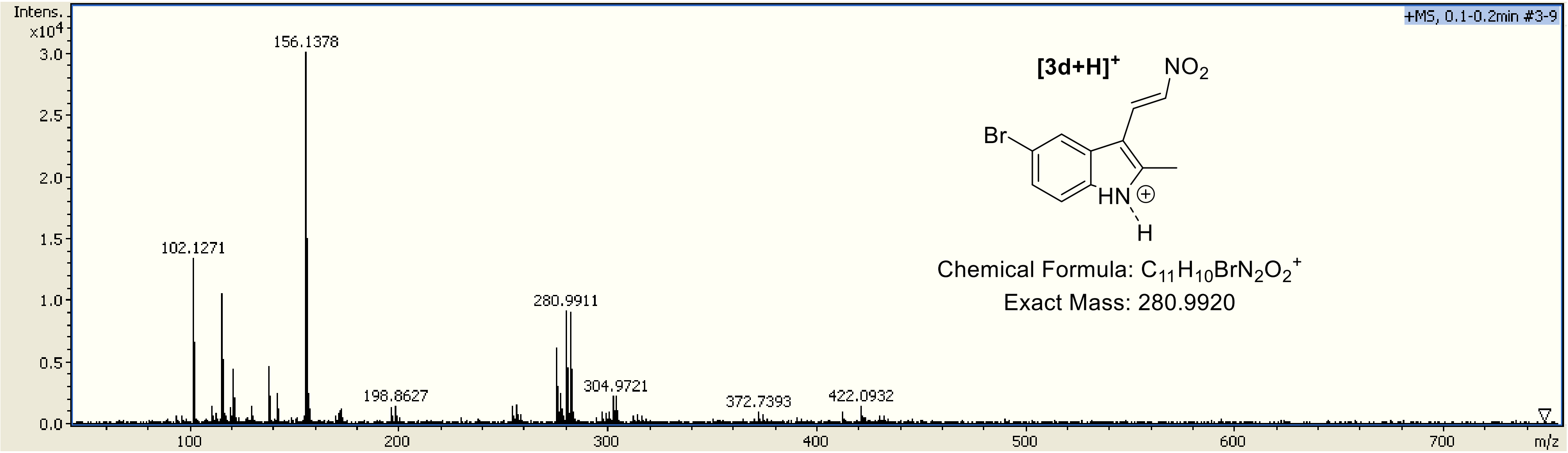

Figure S31. HRMS spectral chart for **3d**

HRMS spectral charts for 3-(1*H*-indol-3-yl)benzofuran-2(3*H*)-ones (8aa-8dd)

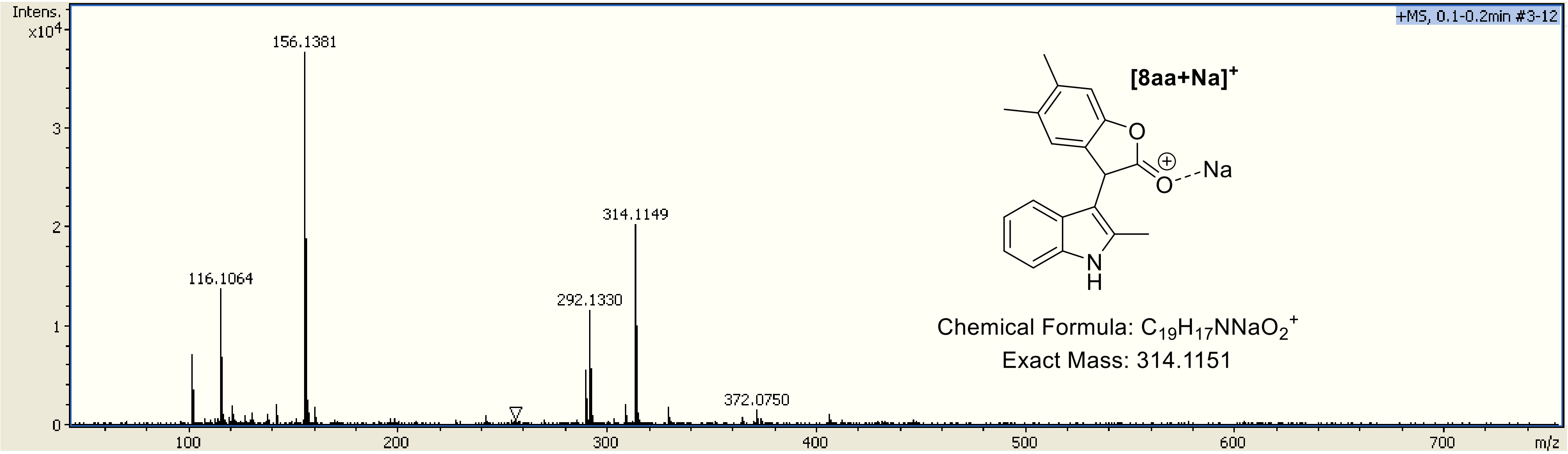

Figure S32. HRMS spectral chart for **8aa**

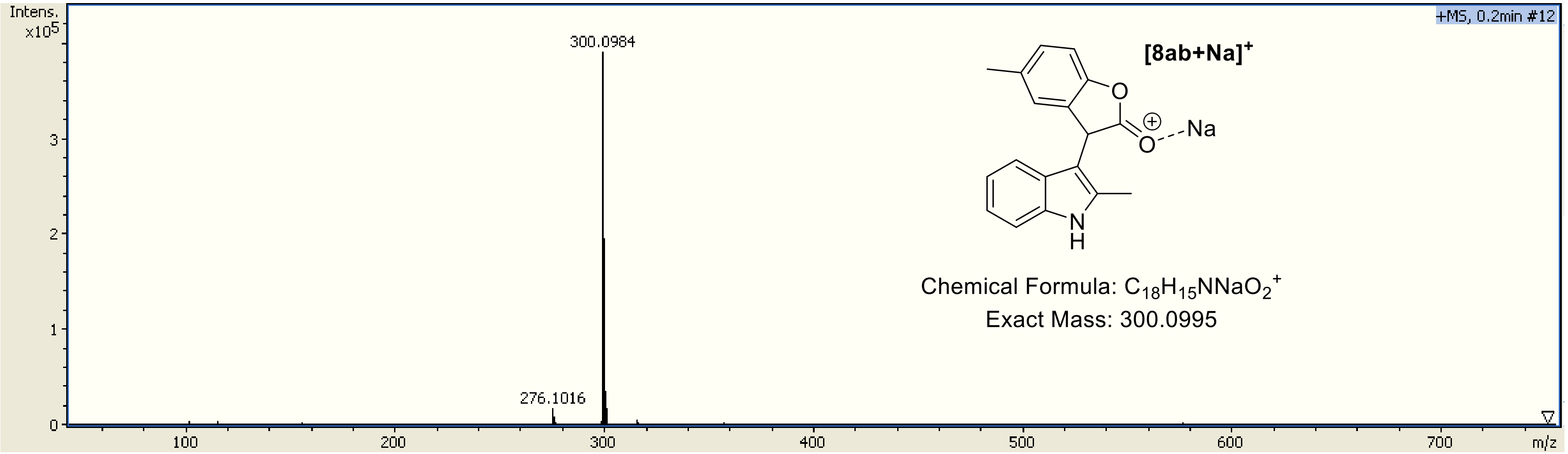

Figure S33. HRMS spectral chart for **8ab**

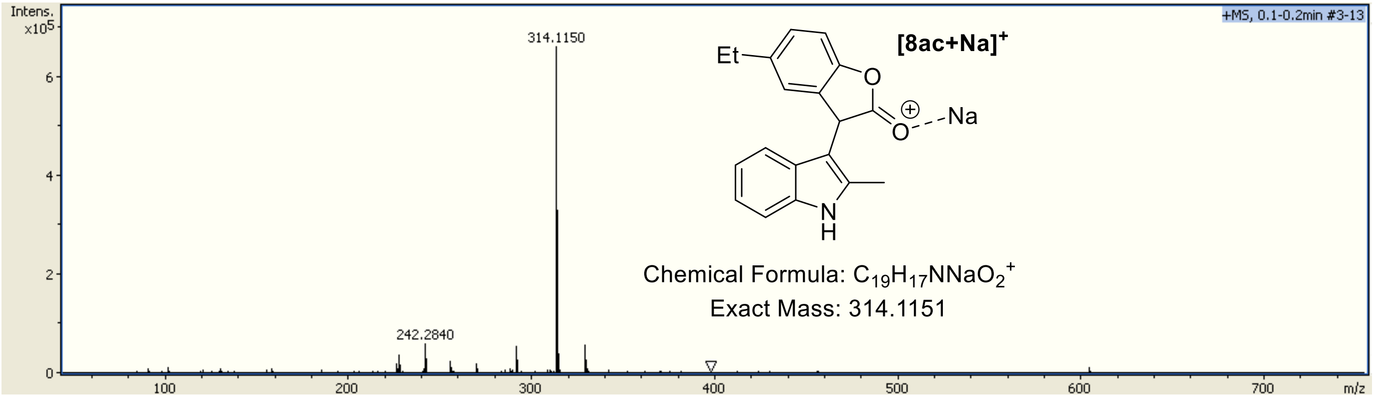

Figure S34. HRMS spectral chart for **8ac**

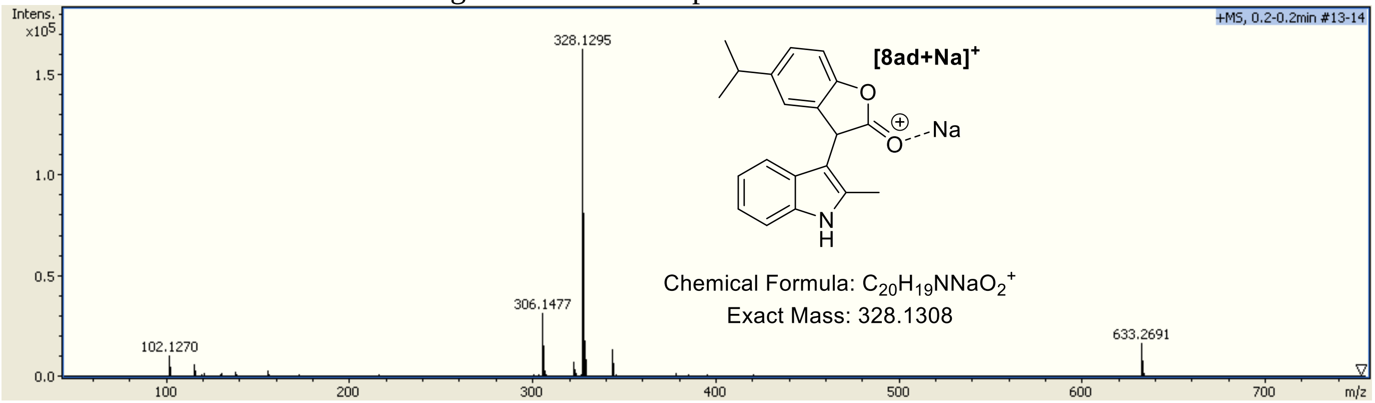

Figure S35. HRMS spectral chart for **8ad**

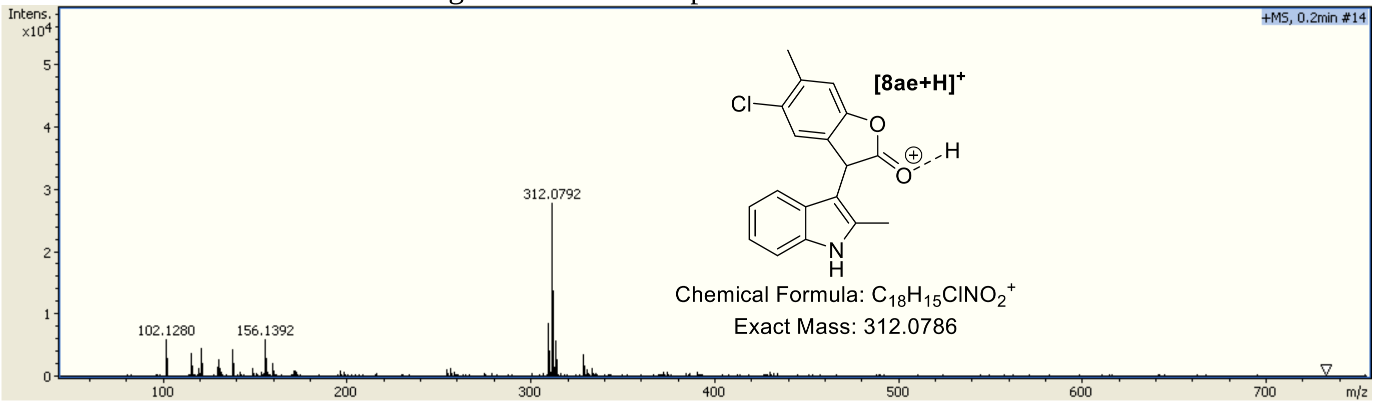

Figure S36. HRMS spectral chart for **8ae**

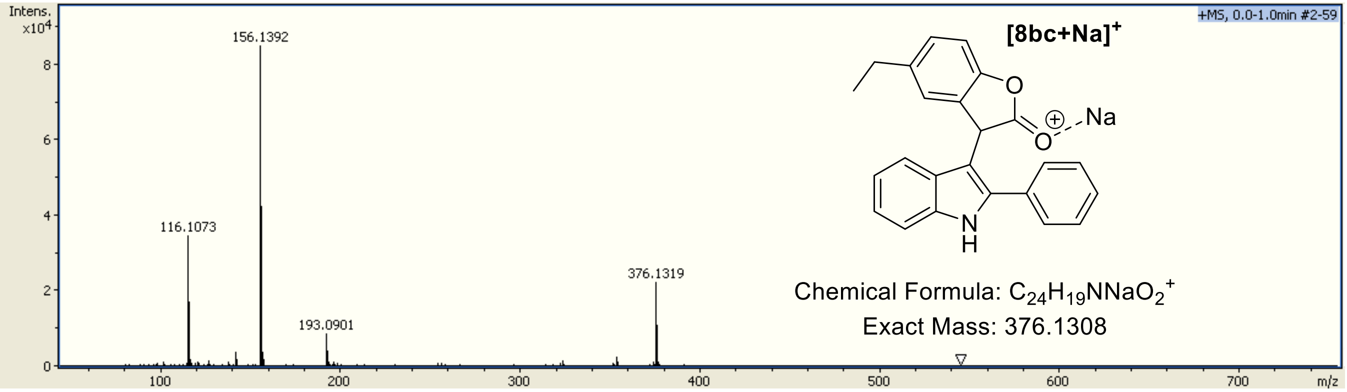

Figure S37. HRMS spectral chart for **8bc**

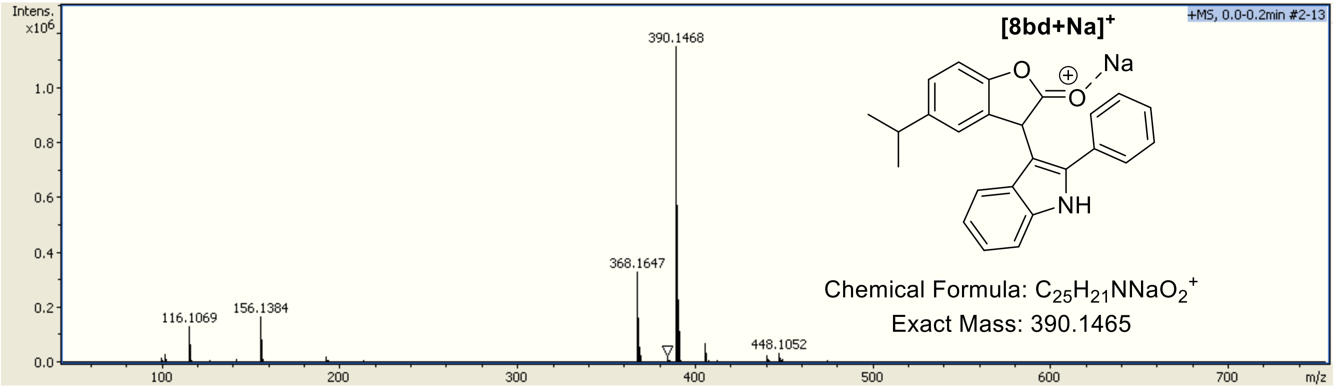

Figure S38. HRMS spectral chart for **8bd**

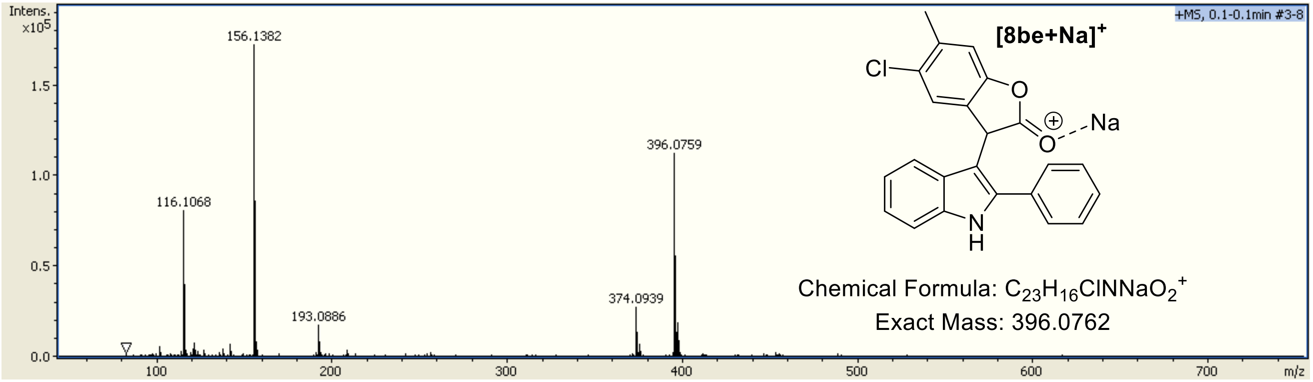

Figure S39. HRMS spectral chart for **8be**

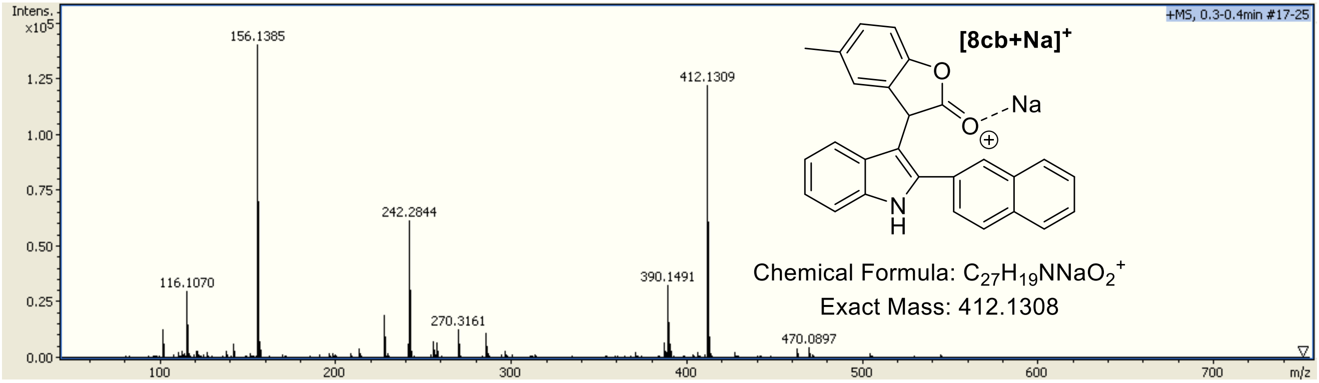

Figure S40. HRMS spectral chart for **8cb**

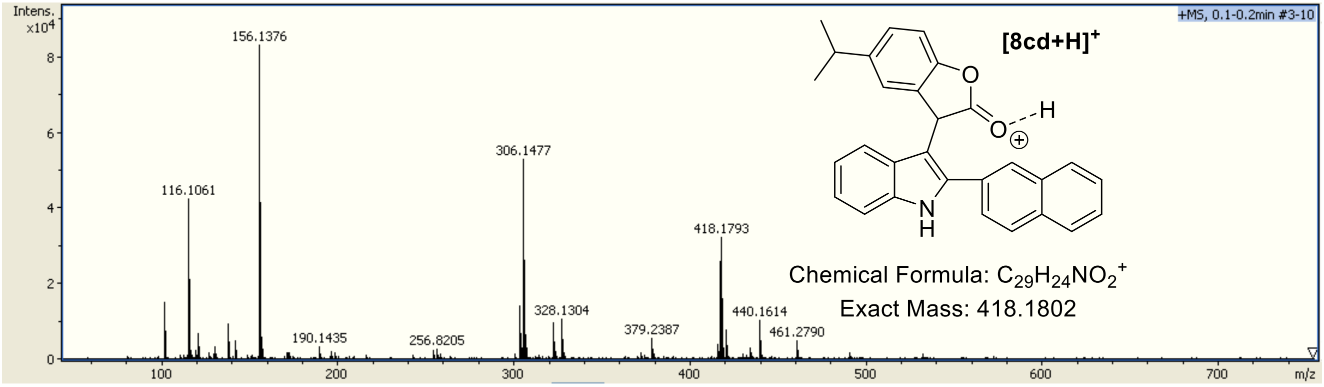

Figure S41. HRMS spectral chart for **8cd**

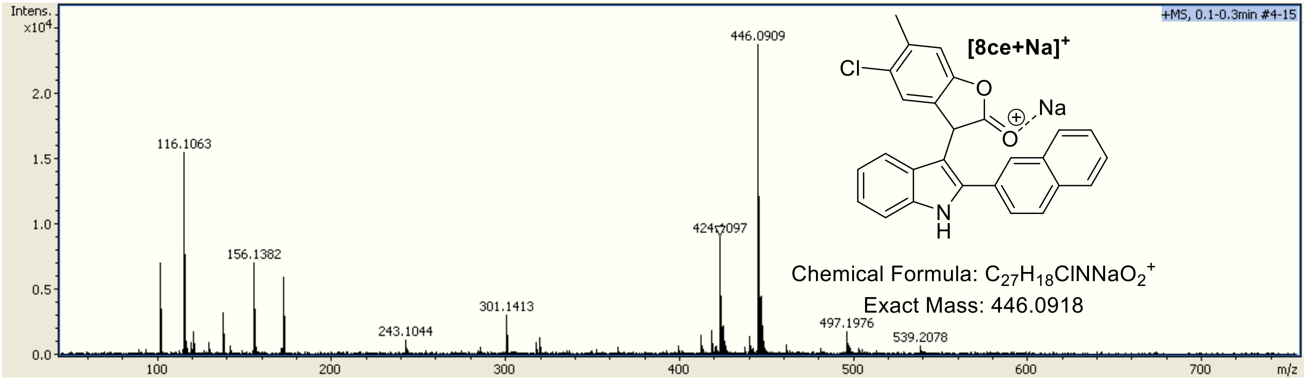

Figure S42. HRMS spectral chart for **8ce**

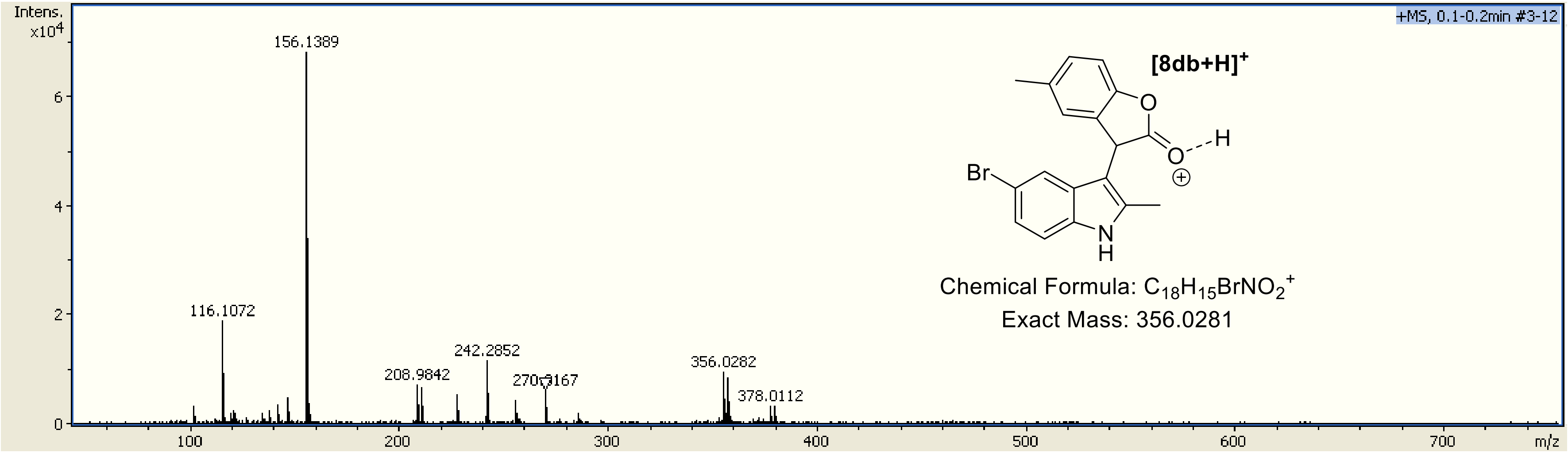

Figure S43. HRMS spectral chart for **8db**

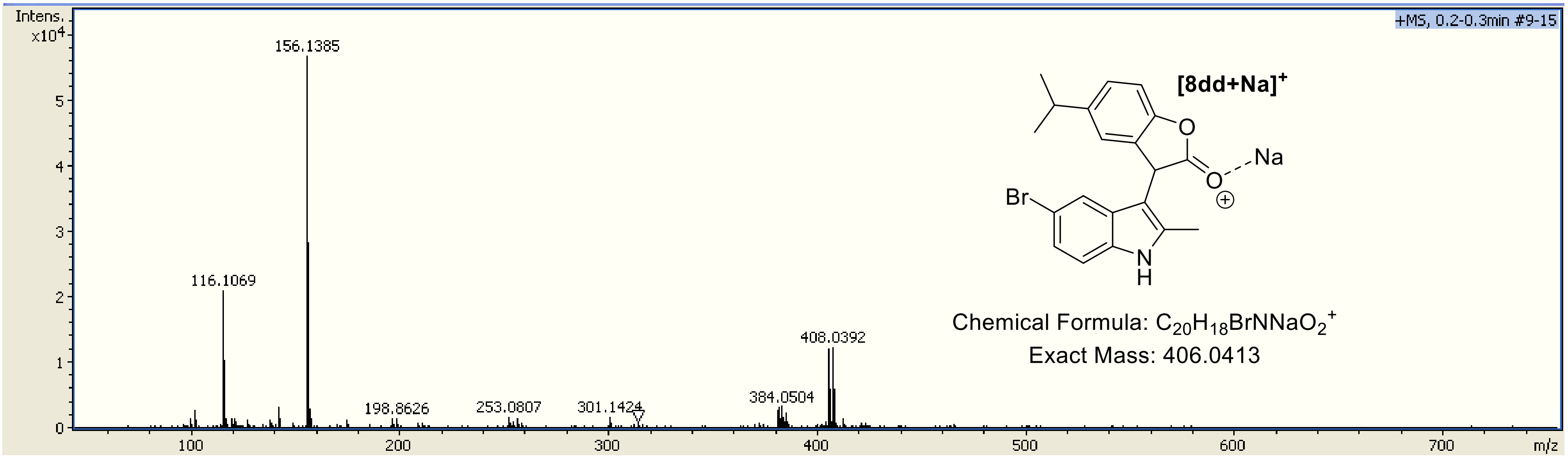

Figure S44. HRMS spectral chart for **8dd**

HRMS spectral chart for 4,5-Dimethyl-2-(1-(2-methyl-1*H*-indol-3-yl)-2-nitroethyl)phenol (9aa):

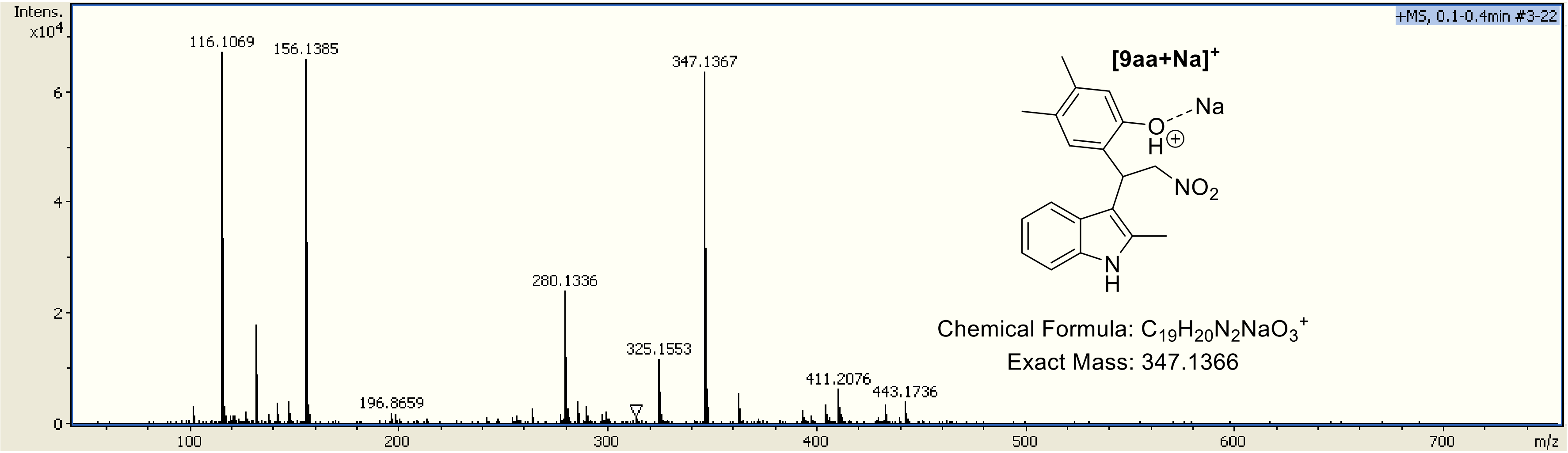

Figure S45. HRMS spectral chart for **9aa**

X-Ray crystallography data

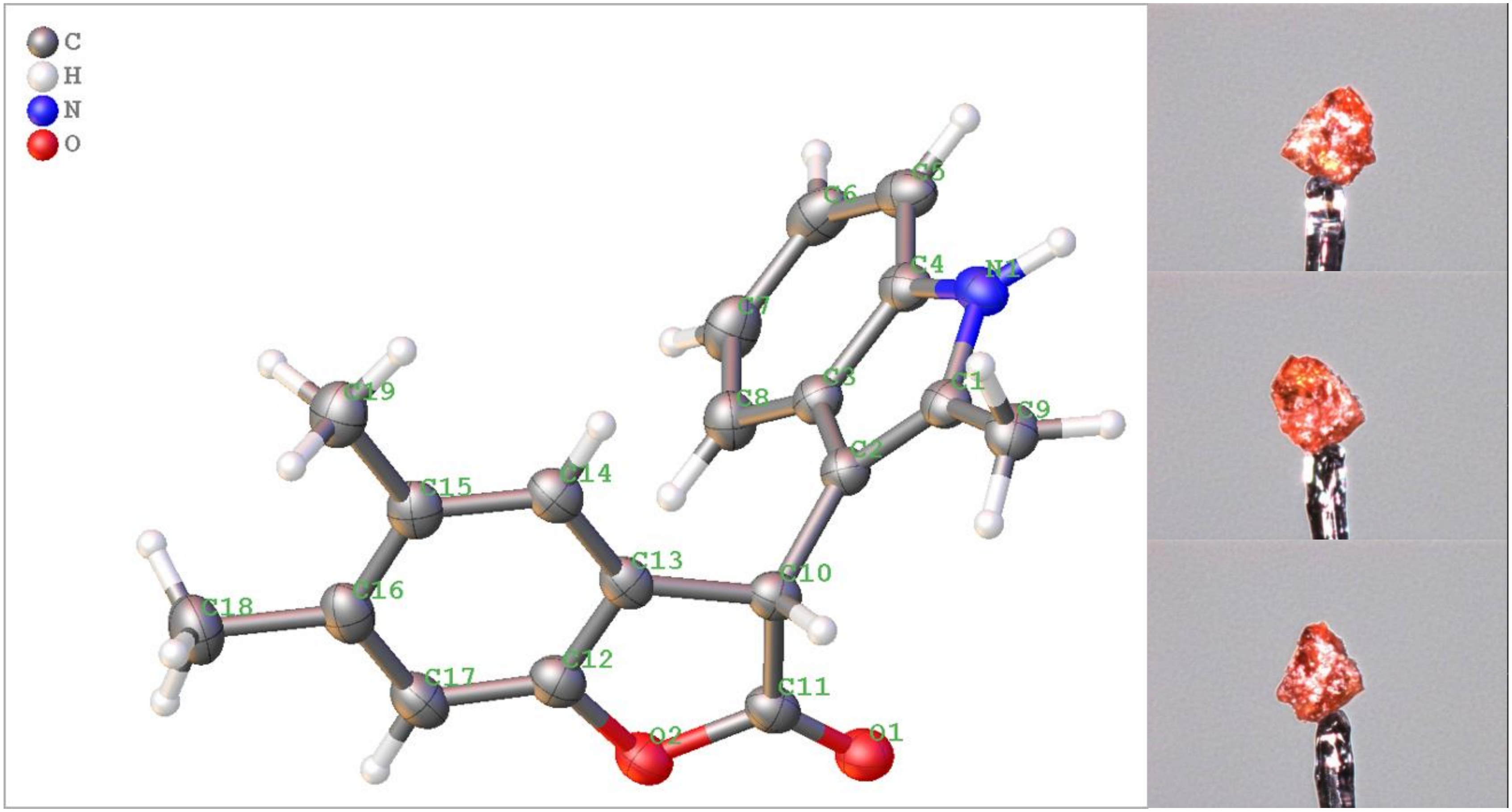

Figure S46. ORTEP drawing of the crystal structure showing 50% probability thermal ellipsoids (left, CCDC 2154293) and microphotography of the single crystal of compound **8aa** used for X-Ray diffraction analysis at the bottom.

Table S1 Crystal data and structure refinement for 8aa.

Identification code	ANNA1909_2
Empirical formula	$C_{19}H_{17}NO_2$
Formula weight	291.33
Temperature/K	100(2)
Crystal system	monoclinic
Space group	$P2_1/c$
a/Å	14.0214(2)
b/Å	12.59540(10)
c/Å	8.61000(10)
α/°	90
β/°	100.8210(10)
γ/°	90
Volume/Å^3	1493.53(3)
Z	4
ρ_{calc}g/cm^3	1.296
μ/mm^{-1}	0.670
F(000)	616.0
Crystal size/mm^3	0.32 × 0.24 × 0.21
Radiation	Cu Kα (λ = 1.54184)
2Θ range for data collection/°	6.418 to 152.188
Index ranges	$-17 \leq h \leq 17$, $-15 \leq k \leq 15$, $-10 \leq l \leq 10$
Reflections collected	25194
Independent reflections	3124 [R_{int} = 0.0270, R_{sigma} = 0.0127]
Data/restraints/parameters	3124/0/206
Goodness-of-fit on F^2	1.038
Final R indexes [I>=2σ (I)]	R_1 = 0.0360, wR_2 = 0.0935
Final R indexes [all data]	R_1 = 0.0381, wR_2 = 0.0954
Largest diff. peak/hole / e Å^{-3}	0.21/-0.18

Table S2 Fractional Atomic Coordinates (×10^4) and Equivalent Isotropic Displacement Parameters (Å^2×10^3) for 8aa. U_{eq} is defined as 1/3 of of the trace of the orthogonalised U_{IJ} tensor.

Atom	*x*	*y*	*z*	U(eq)
O1	8887.8(6)	2418.0(6)	4660.4(10)	33.7(2)
O2	7323.3(6)	2244.9(6)	4828.3(9)	32.67(19)
N1	9335.0(7)	6235.5(7)	5335.5(11)	28.4(2)
C2	8520.0(7)	4727.8(8)	5507.9(12)	24.8(2)
C1	9183.4(7)	5416.4(8)	6327.2(12)	25.8(2)
C3	8252.7(7)	5118.9(8)	3916.9(12)	26.0(2)
C13	7058.2(8)	3755.8(8)	6200.8(12)	27.5(2)
C11	8198.4(8)	2759.0(8)	5147.3(12)	28.2(2)
C10	8124.2(8)	3751.9(8)	6146.8(12)	26.4(2)
C14	6479.3(8)	4456.5(9)	6838.0(12)	29.3(2)
C12	6644.4(8)	2851.7(9)	5458.4(13)	30.1(2)
C4	8781.4(8)	6064.9(8)	3853.9(13)	27.5(2)
C8	7642.0(8)	4753.6(10)	2544.7(13)	31.7(2)
C15	5494.4(8)	4234.2(10)	6758.5(12)	31.6(2)
C9	9687.3(8)	5406.0(9)	8014.4(13)	31.0(2)
C16	5095.8(8)	3295.3(10)	6011.5(13)	34.2(3)
C5	8702.9(9)	6664.6(9)	2478.2(14)	34.1(3)
C17	5680.0(9)	2595.0(10)	5341.4(14)	34.4(3)
C7	7569.2(9)	5346.4(11)	1170.0(14)	37.0(3)
C6	8089.2(9)	6294.3(10)	1142.7(14)	37.5(3)
C19	4872.4(9)	5001.9(11)	7462.8(15)	38.4(3)
C18	4031.9(9)	3046.4(12)	5913.5(17)	44.1(3)

Table S3 Anisotropic Displacement Parameters (Å^2×10^3) for 8aa. The Anisotropic displacement factor exponent takes the form: -2π^2[h^{2}a*^{2}U$_{11}$+2hka*b*U$_{12}$+…].

Atom	U_{11}	U_{22}	U_{33}	U_{23}	U_{13}	U_{12}
O1	36.8(4)	28.5(4)	36.8(4)	-2.3(3)	9.2(3)	-0.4(3)
O2	36.4(4)	27.3(4)	34.7(4)	-3.5(3)	7.7(3)	-5.1(3)
N1	31.9(5)	24.1(4)	31.4(5)	-3.5(3)	11.5(4)	-2.5(4)
C2	26.4(5)	25.2(5)	23.4(5)	0.1(4)	6.4(4)	1.0(4)
C1	27.3(5)	25.0(5)	26.5(5)	-3.2(4)	9.1(4)	2.1(4)
C3	26.5(5)	27.3(5)	25.1(5)	1.2(4)	7.7(4)	2.3(4)
C13	32.8(5)	29.9(5)	19.8(5)	3.5(4)	5.3(4)	-4.5(4)
C11	33.6(5)	25.3(5)	25.5(5)	2.4(4)	4.8(4)	-2.3(4)
C10	31.1(5)	26.2(5)	21.8(5)	0.8(4)	4.2(4)	-1.1(4)
C14	35.1(6)	32.2(5)	21.1(5)	1.0(4)	6.1(4)	-3.5(4)
C12	35.5(6)	29.7(5)	25.9(5)	2.3(4)	7.4(4)	-2.3(4)
C4	30.2(5)	25.5(5)	29.4(5)	-0.6(4)	11.9(4)	3.0(4)
C8	30.1(5)	37.9(6)	26.9(5)	2.0(4)	4.8(4)	-1.5(4)
C15	34.3(6)	37.9(6)	23.1(5)	4.7(4)	6.9(4)	-1.5(5)
C9	31.6(5)	33.3(6)	27.8(5)	-7.3(4)	4.7(4)	-0.2(4)
C16	33.6(6)	40.3(6)	29.0(5)	6.3(5)	6.5(4)	-6.1(5)
C5	40.9(6)	28.4(5)	37.5(6)	6.6(4)	18.8(5)	5.0(5)
C17	38.5(6)	32.9(6)	31.3(6)	0.9(4)	5.4(5)	-9.3(5)
C7	33.9(6)	50.4(7)	26.0(5)	4.4(5)	4.1(4)	5.6(5)
C6	41.5(6)	43.4(7)	30.8(6)	13.6(5)	14.9(5)	13.1(5)
C19	36.2(6)	48.1(7)	32.6(6)	2.2(5)	11.2(5)	0.9(5)
C18	35.2(6)	51.4(8)	46.3(7)	2.4(6)	9.2(5)	-9.0(6)

Table S4 Bond Lengths for 8aa.

Atom	Atom	Length/Å	Atom	Atom	Length/Å
O1	C11	1.2028(14)	C13	C12	1.3793(15)
O2	C11	1.3688(13)	C11	C10	1.5327(14)
O2	C12	1.4067(14)	C14	C15	1.3980(16)
N1	C1	1.3809(14)	C12	C17	1.3755(16)
N1	C4	1.3800(14)	C4	C5	1.3917(15)
C2	C1	1.3669(15)	C8	C7	1.3870(16)
C2	C3	1.4377(14)	C15	C16	1.4106(17)
C2	C10	1.4954(14)	C15	C19	1.5040(17)
C1	C9	1.4914(15)	C16	C17	1.3997(18)
C3	C4	1.4097(15)	C16	C18	1.5112(16)
C3	C8	1.4003(15)	C5	C6	1.3815(18)
C13	C10	1.5042(15)	C7	C6	1.4014(18)
C13	C14	1.3805(16)			

Table S5 Bond Angles for 8aa.

Atom	Atom	Atom	Angle/°	Atom	Atom	Atom	Angle/°
C11	O2	C12	108.16(8)	C13	C10	C11	101.11(8)
C4	N1	C1	109.27(9)	C13	C14	C15	120.11(10)
C1	C2	C3	107.74(9)	C13	C12	O2	111.78(10)
C1	C2	C10	126.53(9)	C17	C12	O2	124.84(10)
C3	C2	C10	125.72(9)	C17	C12	C13	123.38(11)
N1	C1	C9	120.60(9)	N1	C4	C3	107.74(9)
C2	C1	N1	108.89(9)	N1	C4	C5	130.03(10)
C2	C1	C9	130.48(10)	C5	C4	C3	122.23(11)
C4	C3	C2	106.34(9)	C7	C8	C3	118.59(11)
C8	C3	C2	134.43(10)	C14	C15	C16	119.56(11)
C8	C3	C4	119.21(10)	C14	C15	C19	119.59(11)
C14	C13	C10	132.55(10)	C16	C15	C19	120.85(11)

Table S5 Bond Angles for 8aa.

Atom	Atom	Atom	Angle/°	Atom	Atom	Atom	Angle/°
C12	C13	C10	108.48(9)	C15	C16	C18	120.12(11)
C12	C13	C14	118.97(10)	C17	C16	C15	120.25(10)
O1	C11	O2	120.66(10)	C17	C16	C18	119.62(11)
O1	C11	C10	129.11(10)	C6	C5	C4	117.55(11)
O2	C11	C10	110.23(9)	C12	C17	C16	117.71(11)
C2	C10	C13	116.63(9)	C8	C7	C6	121.18(11)
C2	C10	C11	113.28(9)	C5	C6	C7	121.22(11)

Table S6 Torsion Angles for 8aa.

A	B	C	D	Angle/°	A	B	C	D	Angle/°
O1	C11	C10	C2	49.60(15)	C10	C2	C3	C4	178.47(9)
O1	C11	C10	C13	175.17(11)	C10	C2	C3	C8	-3.30(19)
O2	C11	C10	C2	-130.43(9)	C10	C13	C14	C15	-177.68(10)
O2	C11	C10	C13	-4.86(11)	C10	C13	C12	O2	-2.77(12)
O2	C12	C17	C16	-178.91(10)	C10	C13	C12	C17	178.04(10)
N1	C4	C5	C6	179.49(10)	C14	C13	C10	C2	-53.04(15)
C2	C3	C4	N1	-0.27(11)	C14	C13	C10	C11	-176.34(11)
C2	C3	C4	C5	179.94(10)	C14	C13	C12	O2	177.89(9)
C2	C3	C8	C7	-178.89(11)	C14	C13	C12	C17	-1.30(16)
C1	N1	C4	C3	0.93(11)	C14	C15	C16	C17	-0.58(16)
C1	N1	C4	C5	-179.30(11)	C14	C15	C16	C18	-179.85(11)
C1	C2	C3	C4	-0.49(11)	C12	O2	C11	O1	-176.53(10)
C1	C2	C3	C8	177.73(12)	C12	O2	C11	C10	3.50(11)

Table S6 Torsion Angles for 8aa.

A	B	C	D	Angle/°	A	B	C	D	Angle/°
C1	C2	C10	C13	114.60(12)	C12	C13	C10	C2	127.73(10)
C1	C2	C10	C11	-128.64(11)	C12	C13	C10	C11	4.44(11)
C3	C2	C1	N1	1.07(12)	C12	C13	C14	C15	1.47(15)
C3	C2	C1	C9	178.95(10)	C4	N1	C1	C2	-1.26(12)
C3	C2	C10	C13	-64.17(14)	C4	N1	C1	C9	-179.39(9)
C3	C2	C10	C11	52.59(14)	C4	C3	C8	C7	-0.84(16)
C3	C4	C5	C6	-0.77(16)	C4	C5	C6	C7	-0.37(17)
C3	C8	C7	C6	-0.27(18)	C8	C3	C4	N1	-178.82(9)
C13	C14	C15	C16	-0.56(16)	C8	C3	C4	C5	1.39(16)
C13	C14	C15	C19	179.76(10)	C8	C7	C6	C5	0.90(18)
C13	C12	C17	C16	0.17(17)	C15	C16	C17	C12	0.77(17)
C11	O2	C12	C13	-0.48(12)	C19	C15	C16	C17	179.10(10)
C11	O2	C12	C17	178.69(10)	C19	C15	C16	C18	-0.18(17)
C10	C2	C1	N1	-177.88(9)	C18	C16	C17	C12	-179.96(11)
C10	C2	C1	C9	-0.01(18)					

Table S7 Hydrogen Atom Coordinates (Å×10^4) and Isotropic Displacement Parameters (Å^2×10^3) for 8aa.

Atom	*x*	*y*	*z*	U(eq)
H10	8495.94	3622.09	7239.98	32
H14	6750.59	5090.68	7331.16	35
H8	7285.22	4113.28	2555.25	38
H9A	10381.1	5543.99	8073.66	47
H9B	9602.03	4710.08	8478.64	47
H9C	9410.03	5957.16	8599.44	47
H5	9058.26	7305.06	2458.17	41
H17	5419.45	1964.16	4823.58	41

Table S7 Hydrogen Atom Coordinates (Å×10^4) and Isotropic Displacement Parameters (Å^2×10^3) for 8aa.

Atom	*x*	*y*	*z*	U(eq)
H7	7159.56	5105.55	229.94	44
H6	8018.33	6689.59	188.19	45
H19A	5272.29	5596.3	7945.94	58
H19B	4585.14	4639.23	8271.03	58
H19C	4354.29	5270.46	6630.2	58
H18A	3899.61	2945.08	6981.6	66
H18B	3867.12	2396.2	5296.16	66
H18C	3638.89	3636.41	5399.22	66
H1	9865(11)	6696(12)	5526(17)	42(4)

Experimental

Single crystals of $C_{19}H_{17}NO_2$ **8aa** were otained by slow evaporation of saturated solution in EtOAc. A suitable crystal was selected and mounted on the glass stick by acrylic glue on a **SuperNova, Dual, Cu at home/near, AtlasS2** diffractometer. The crystal was kept at 100(2) K during data collection. Using Olex2 [1], the structure was solved with the SHELXT [2] structure solution program using Intrinsic Phasing and refined with the SHELXL [3] refinement package using Least Squares minimisation.

Crystal structure determination of 8aa

Crystal Data for $C_{19}H_{17}NO_2$ (M =291.33 g/mol): monoclinic, space group P2_1/c (no. 14), a = 14.0214(2) Å, b = 12.59540(10) Å, c = 8.61000(10) Å, β = 100.8210(10)°, V = 1493.53(3) Å^3, Z = 4, T = 100(2) K, μ(Cu Kα) = 0.670 mm^{-1}, $Dcalc$ = 1.296 g/cm^3, 25194 reflections measured (6.418° ≤ 2Θ ≤ 152.188°), 3124 unique (R_{int} = 0.0270, R_{sigma} = 0.0127) which were used in all calculations. The final R_1 was 0.0360 (I > 2σ(I)) and wR_2 was 0.0954 (all data).

Refinement model description

Number of restraints - 0, number of constraints - 0.

Details:

```
1. Fixed Uiso
 At 1.2 times of:
  All C(H) groups
 At 1.5 times of:
  All C(H,H,H) groups
2.a Ternary CH refined with riding coordinates:
 C10(H10)
2.b Aromatic/amide H refined with riding coordinates:
 C14(H14), C8(H8), C5(H5), C17(H17), C7(H7), C6(H6)
2.c Idealised Me refined as rotating group:
 C9(H9A,H9B,H9C), C19(H19A,H19B,H19C), C18(H18A,H18B,H18C)
```

This report has been created with Olex2, compiled on 2020.11.12 svn.r5f609507 for OlexSys. Please let us know if there are any errors or if you would like to have additional features.

References

1. Dolomanov, O.V., Bourhis, L.J., Gildea, R.J, Howard, J.A.K. & Puschmann, H. (2009), *J. Appl. Cryst*. 42, 339-341.
2. Sheldrick, G.M. (2008). *Acta Cryst*. A64, 112-122.
3. Sheldrick, G.M. (2015). *Acta Cryst*. C71, 3-8.
