# Supplementary material for: Improved Method for Preparation of 3-(1H-Indol-3-yl)benzofuran-2(3H)-ones
Source: Molecules. 2022 Mar 15;27(6):1902. doi: 10.3390/molecules27061902 (PMC8950327; doi:10.3390/molecules27061902)

checkCIF/PLATON report

Structure factors have been supplied for datablock(s) anna1909_2

THIS REPORT IS FOR GUIDANCE ONLY. IF USED AS PART OF A REVIEW PROCEDURE FOR PUBLICATION, IT SHOULD NOT REPLACE THE EXPERTISE OF AN EXPERIENCED CRYSTALLOGRAPHIC REFEREE.

No syntax errors found. CIF dictionary Interpreting this report

Datablock: anna1909_2

```
Bond precision:   C-C = 0.0016 A          Wavelength=1.54184

Cell:             a=14.0214(2)     b=12.5954(1)        c=8.6100(1)
                  alpha=90         beta=100.821(1)     gamma=90
Temperature:      100 K
```

	Calculated	Reported
Volume	1493.53(3)	1493.53(3)
Space group	P 21/c	P 1 21/c 1
Hall group	-P 2ybc	-P 2ybc
Moiety formula	C19 H17 N O2	C19 H17 N O2
Sum formula	C19 H17 N O2	C19 H17 N O2
Mr	291.34	291.33
Dx,g cm-3	1.296	1.296
Z	4	4
Mu (mm-1)	0.670	0.670
F000	616.0	616.0
F000'	617.80	
h,k,lmax	17,15,10	17,15,10
Nref	3124	3124
Tmin,Tmax	0.825,0.869	0.914,0.942
Tmin'	0.807	

```
Correction method= # Reported T Limits: Tmin=0.914 Tmax=0.942
AbsCorr = ANALYTICAL

Data completeness= 1.000          Theta(max)= 76.094

R(reflections)= 0.0360( 2924)     wR2(reflections)= 0.0954( 3124)

S = 1.038                 Npar= 206
```

The following ALERTS were generated. Each ALERT has the format
test-name_ALERT_alert-type_alert-level.
Click on the hyperlinks for more details of the test.

Alert level A

```
EXPT005_ALERT_1_A  _exptl_crystal_description is missing
            Crystal habit description.
            The following tests will not be performed.
            CRYSR_01
PLAT699_ALERT_1_A Missing _exptl_crystal_description Value .......     Please Do !
```

Alert level G

```
PLAT142_ALERT_4_G s.u. on b - Axis Small or Missing .............    0.00010 Ang.
PLAT398_ALERT_2_G Deviating  C-O-C   Angle From 120 for O2       .     108.2 Degree
PLAT793_ALERT_4_G Model has Chirality at C10         (Centro SPGR)         S Verify
PLAT912_ALERT_4_G Missing # of FCF Reflections Above STh/L=  0.600         1 Note
PLAT978_ALERT_2_G Number C-C Bonds with Positive Residual Density.        17 Info
```

2 **ALERT level A** = Most likely a serious problem - resolve or explain
0 **ALERT level B** = A potentially serious problem, consider carefully
0 **ALERT level C** = Check. Ensure it is not caused by an omission or oversight
5 **ALERT level G** = General information/check it is not something unexpected

2 ALERT type 1 CIF construction/syntax error, inconsistent or missing data
2 ALERT type 2 Indicator that the structure model may be wrong or deficient
0 ALERT type 3 Indicator that the structure quality may be low
3 ALERT type 4 Improvement, methodology, query or suggestion
0 ALERT type 5 Informative message, check

It is advisable to attempt to resolve as many as possible of the alerts in all categories. Often the minor alerts point to easily fixed oversights, errors and omissions in your CIF or refinement strategy, so attention to these fine details can be worthwhile. In order to resolve some of the more serious problems it may be necessary to carry out additional measurements or structure refinements. However, the purpose of your study may justify the reported deviations and the more serious of these should normally be commented upon in the discussion or experimental section of a paper or in the "special_details" fields of the CIF. checkCIF was carefully designed to identify outliers and unusual parameters, but every test has its limitations and alerts that are not important in a particular case may appear. Conversely, the absence of alerts does not guarantee there are no aspects of the results needing attention. It is up to the individual to critically assess their own results and, if necessary, seek expert advice.

Publication of your CIF in IUCr journals

A basic structural check has been run on your CIF. These basic checks will be run on all CIFs submitted for publication in IUCr journals (*Acta Crystallographica*, *Journal of Applied Crystallography*, *Journal of Synchrotron Radiation*); however, if you intend to submit to *Acta Crystallographica Section C* or *E* or *IUCrData*, you should make sure that full publication checks are run on the final version of your CIF prior to submission.

Publication of your CIF in other journals

Please refer to the *Notes for Authors* of the relevant journal for any special instructions relating to CIF submission.

PLATON version of 19/02/2022; check.def file version of 19/02/2022

Datablock anna1909_2 - ellipsoid plot

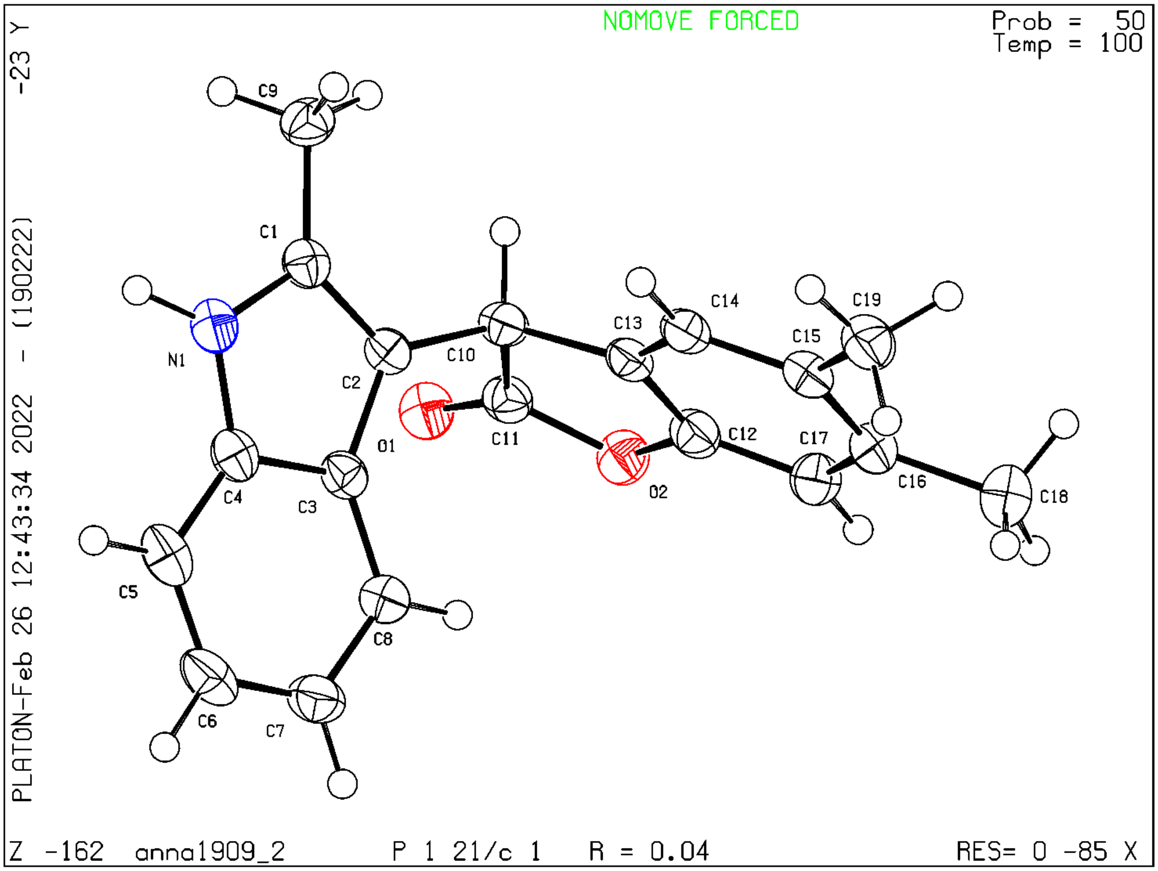

Supplement: Supplementary file 1 [file molecules-27-01902-s001.zip › suppl/checkcif.pdf]
